# Temperature Dependence of Structural Relaxation in Glass-Forming Liquids and Polymers

**DOI:** 10.3390/e24081101

**Published:** 2022-08-10

**Authors:** Vladimir N. Novikov, Alexei P. Sokolov

**Affiliations:** 1 Institute of Automation and Electrometry, Russian Academy of Sciences, 630090 Novosibirsk, Russia; 2 Department of Chemistry and Joint Institute for Neutron Sciences, University of Tennessee, Knoxville, TN 37996, USA; 3 Chemical Sciences Division, Oak Ridge National Laboratory, Oak Ridge, TN 37831, USA

**Keywords:** glass transition, fragility, supercooled liquids, relaxation

## Abstract

Understanding the microscopic mechanism of the transition of glass remains one of the most challenging topics in Condensed Matter Physics. What controls the sharp slowing down of molecular motion upon approaching the glass transition temperature *T_g_*, whether there is an underlying thermodynamic transition at some finite temperature below *T_g_*, what the role of cooperativity and heterogeneity are, and many other questions continue to be topics of active discussions. This review focuses on the mechanisms that control the steepness of the temperature dependence of structural relaxation (fragility) in glass-forming liquids. We present a brief overview of the basic theoretical models and their experimental tests, analyzing their predictions for fragility and emphasizing the successes and failures of the models. Special attention is focused on the connection of fast dynamics on picosecond time scales to the behavior of structural relaxation on much longer time scales. A separate section discusses the specific case of polymeric glass-forming liquids, which usually have extremely high fragility. We emphasize the apparent difference between the glass transitions in polymers and small molecules. We also discuss the possible role of quantum effects in the glass transition of light molecules and highlight the recent discovery of the unusually low fragility of water. At the end, we formulate the major challenges and questions remaining in this field.

## 1. Introduction

Glass transition is a transition from a liquid to a solid state upon cooling without crystallization. There are many liquids that can be easily supercooled below their melting temperature *T_m_* and will form a solid glassy state at the corresponding glass transition temperature *T_g_*. The latter is defined as the temperature at which the structural relaxation time *τ_α_* reaches ~10^2^–10^3^ s. In that respect, the transition at *T_g_* is not a phase transition but has a kinetic origin, i.e., marks the transition to a nonequilibrium state when the structural relaxation rate becomes comparable to the experimental cooling rate. Despite many decades of studies, understanding the microscopic mechanism of the sharp increase in *τ_α_* or viscosity *η* upon approaching the glass transition remains a great challenge [1,2,3,4,5,6]. This understanding is of critical importance not only for the field of glass transition but also for many other areas, including general Soft Matter Physics and Biophysics. Glass transition can be considered as a ‘model phenomenon’ that provides a microscopic picture of complex dynamics with significant cooperativity and dynamic heterogeneity in molecular motions. Understanding the dynamics of glass-forming systems can be extrapolated to even more complex systems (e.g., proteins).

**Figure 1:**
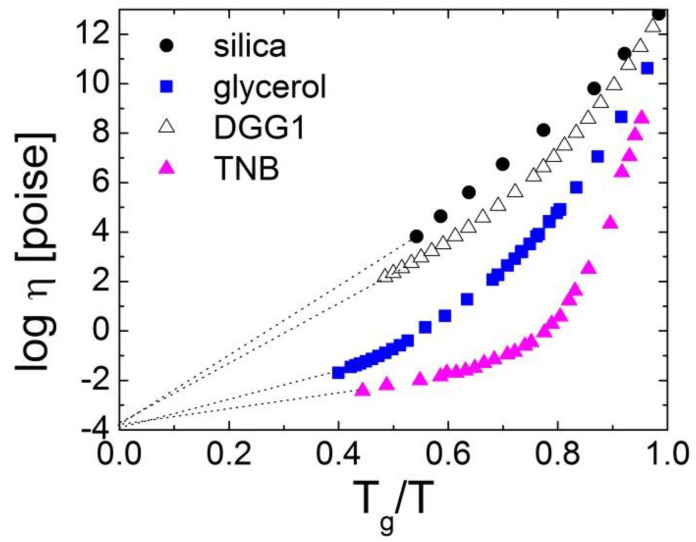
The dependence of the viscosity in some glass-forming liquids on *T_g_*/*T*. Here, TNB is trisnaphthyl benzene, and DGG1 is a soda lime silica glass. Data from Ref. [14] and the references therein.

In all liquids at high enough temperatures, the viscosity *η*(*T*) or structural relaxation time *τ_α_*(*T*) exhibit Arrhenius behavior with some constant activation energy *E*_∞_. At lower temperatures, however, they exhibit super-Arrhenius behavior: *η*(*T*) and/or *τ_α_*(*T*) change much faster than they would for a simple Arrhenius law (Figure 1), reflecting an increase in the activation energy for structural relaxation. The deviation from the Arrhenius behavior is highly dependent on the liquid and is usually weak for covalent bonding systems (e.g., SiO_2_) and strong for Van der Waals (VdW) liquids (e.g., trisnaphtyl benzene) (Figure 1). The degree of non-Arrhenius behavior is characterized by the steepness of the temperature variation of *η*(*T*) or *τ_α_*(*T*) at *T_g_*. It is traditionally called fragility [1,7,8] and is defined as:

(1) $$m={\left.\frac{d\log\eta }{dT_{g}/T}\right|}_{T=T_{g}}$$

The fragility (steepness) index *m* reflects the apparent activation energy at *T_g_* (with a coefficient ln10) normalized by *T_g_*. We note that different behaviors of viscosities as a function of *T_g_*/*T* were discussed initially by Oldekop, W. [9]. The classification of glass formers according to the rate of the increase in the viscosity close to the glass transition was also suggested by Nemilov, S. [10]. The *T_g_*-scaled Arrhenius plot was reintroduced by Laughlin and Uhlmann [11]. However, the notion of fragility and its definition (Equation (1)) were introduced by A. Angell, and he showed its fundamental importance in supercooled liquids and glass transition [7,8]. With a decreasing temperature, the viscosity of liquids increases from the universal high temperature limit *η*_∞_ of about 10^−4^ Poise [1,12,13] to ~10^13^ Poise at *T_g_*. If it exhibited Arrhenius behavior all the way from the high temperatures to *T_g_*, this would correspond to the lowest possible fragility of *m*_0_ = 17. The lowest known fragility, however, is *m*~18–22 and is found in covalent bonding systems SiO_2_ and BeF_2_ [1,5]. It means that, even in these liquids, the temperature dependence of *η*(*T*) or *τ_α_*(*T*) does not show purely Arrhenius behavior. VdW and ionic liquids exhibit much higher fragility of *m*~80–100 [5], while many polymers have the highest fragility of *m*~150–200 [5,6], showing an extremely steep non-Arrhenius temperature dependence of their structural (segmental) relaxation.

The mechanism of these steep temperature variations in the structural relaxation time (fragility) remains a great puzzle and is the focus of the current review. We emphasize that this puzzle is convoluted, with many fundamental questions of the glass transition effect, such as the extent of the collective dynamics and dynamical heterogeneity, decoupling of various dynamic processes, and connections between the slow and fast dynamics and between the elastic and thermodynamic properties in glass-forming materials. We attempt to address most of these questions in this review. Section 2 presents briefly the major theoretical approaches proposed to describe the super-Arrhenius temperature variations of *τ_α_*(*T*) close to *T_g_*. They can be divided into three main classes: free volume, entropic, and elastic models. Section 3 presents comparisons of experimental data to theoretical predictions. Although all approaches have some problems, they capture many peculiarities of the glass transition, and we attempt to find underlying connections between different approaches. Section 4 discusses a puzzling correlation between the dynamics on a picosecond time scale and the fragility of glass-forming liquids and attempts to formulate some general picture. Section 5 discusses the extremely high fragility of polymers and the role of intramolecular degrees of freedom in their peculiar behavior. Section 6 discusses the possibility of extremely low fragility due to quantum effects and, in particular, focuses on the dynamics of water that exhibit unusually low fragility, *m*~14. Section 7 summarizes the discussion and emphasizes the major challenges in the field of glass transition.

## 2. Theoretical Concepts Proposed for Describing the Temperature Dependence of Structural Relaxation in Supercooled Liquids

There are many theories and models proposed for describing the temperature dependence of the viscosity or structural relaxation time in supercooled liquids. Here, we briefly review the most common and successful models, focusing on their predictions for fragility. Most of them can be classified into one of three major classes: (i) free volume, (ii) elastic models, and (iii) thermodynamic (entropy) models. The success of all these approaches emphasizes that they might reflect different projections of the same underlying mechanism. The possible relationship of these approaches will be discussed. We want to emphasize that, due to volume limitations, we cannot include a comprehensive overview of all the theories. One of the successful and controversial theories of glass transition is the mode-coupling theory (MCT), which offers an interesting insight into the high temperature behavior of glass-forming liquids [15]. However, it focuses mostly on the temperature range above the so-called MCT crossover temperature *T_c_* and does not provide a good description of the dynamics close to *T_g_*, which is the focus of our review. Thus, we will not discuss it here.

### 2.1. Semi-Empirical Equations Describing τ_α_(T)

There are many phenomenological and empirical equations that describe well the temperature variations of structural relaxation *τ_α_*(*T*) or viscosity *η*(*T*) in a broad temperature range. The most traditional is the Vogel-Fulcher-Tammann (VFT) equation [16,17,18]:

*τ*(*Τ*) *= τ*_0_ exp[*B*/(*T − T*_0_)] (2)

where *τ*_0_ is the high temperature limit of *τ, B* and *T*_0_ are the material-dependent parameters. In the polymer science community, Equation (2) is also known as the Williams–Landel–Ferry (WLF) equation [19]:

(3) $$\log\left[\tau (T*)/\tau (T)\right]=\frac{C_{1}\left(T-T*\right)}{T-\left(T*-C_{2}\right)}$$

where T* is some reference temperature, and *C*_1_ and *C*_2_ are the material-dependent constants. It is identical to Equation (2.1), with *C*_2_ = T* − *T*_0_, *B* = *C*_1_*C*_2_ln10, and *C*_1_(T*) = log[*τ*(T*)/*τ*_0_]. The fragility estimated with the VFT function is equal to

(4) $$m=\frac{m_{0}}{1-T_{0}/T_{g}}=\frac{T_{g}m_{0}^{2}\ln10}{B}$$

Here, *m*_0_ = 17 is the fragility that corresponds to purely Arrhenius dynamics. Thus, the fragility increases as *T*_0_ is approaching *T_g_*. In some glass formers (polymers, hydrogen-bonding materials, and room temperature ionic liquids), a single VFT function fits the experimental data fairly well in the entire temperature range. However, in most molecular liquids, two different VFT functions (one at a higher *T* and another at a lower *T*) are needed to describe *η*(*T*) or *τ*(*T*) over the entire temperature range [20].

Several other three-parameter empirical functions have been proposed to describe *τ_α_*(*T*). Avramov and Milchev [21] proposed a model based on thermally activated atomic hopping over barriers with a distribution of barrier heights to justify the empirically found dependence [22,23,24]:

log*τ_α_* = log*τ*_0_ + *B*/*T*^α^ (5)

In this model,

*m* = *αm*_0_ (6)

Thus, the exponent *α* changes from 1 to >8 for various glass formers. As it is typical for the three-parameter models, this fitting function gives a good fit only in a limited temperature range of supercooled liquids.

Mauro et al. recently suggested a model [25] explaining another three-parameter function, which was first proposed to fit the experimental viscosity data [26]. It is based on the temperature dependence of the configurational entropy, which is associated with the topological degrees of freedom per atom. For a simple two-state model, they obtained a double-Arrhenius expression for the *α*-relaxation time:

(7) $$\log(\tau_{\alpha }/\tau_{0})=\frac{K}{T}\exp\left(\frac{E}{T}\right)$$

In the Mauro model, the fragility is determined by the second activation energy *E*:

*m* = *m*_0_ (1 + *E*/*T*_g_) (8)

The larger *E* is in comparison with *T_g_*, the higher the fragility.

Recently, Rössler and coworkers suggested another effective three-parameter function that describes *τ_α_* in liquids over a broad temperature range [27]. They assumed that the total activation energy is the sum of two contributions: *E*(*T*) = *E*_∞_ + *E_coop_*(*T*). *E*_∞_ is the high-temperature activation energy, which does not vary with *T*, while the activation energy of cooperative relaxation, *E_coop_*(*T*), is assumed to vary exponentially with *T*:

*E_coop_*(*T*) ∝ exp[−*λ*(*T*/*T_A_* − 1)] (9)

Here, *T*_*A*_ has the meaning of the temperature above which the relaxation time is purely Arrhenius with the activation energy *E*_∞_. This corresponds to the four-parameter function:

(10) $$\log(\tau_{\alpha }/\tau_{0})=\frac{E_{\infty }+a\exp\left(-\lambda (T/T_{A}-1\right)}{T}$$

At sufficiently low *T*, *T* << *T_A_*/*λ*, this function predicts the Arrhenius temperature dependence of *τ_α_*. Comparison with the experimental data showed that most molecular and hydrogen-bonded systems have *a* ≅ *E*_∞_ and *T_A_* = 0.104*E*_∞_, effectively reducing the number of the parameters to three. The fragility is equal to

*m* = *m*_0_ + *λ*(*m*_0_*T*_g_ − *E*_∞_)/*T_A_* (11)

It is important to emphasize that the VFT (or WLF) function predicts the divergence of the relaxation time at some finite temperature *T*_0_, suggesting an underlying phase transition at *T* below *T_g_*. However, Equations (5), (7) and (10) predict that the relaxation time will not diverge at any finite temperature, and no phase transition is required. This is a longstanding question that still remains unresolved: Is the apparent divergence of *τ_α_*(*T*) real, and is there an ideal glass transition at a finite temperature *T*_0_, or is this divergence a fictitious consequence of the approximations made? A recent analysis of 42 supercooled liquids close to the glass transition claimed no experimental basis for the dynamical divergence of the VFT form [28]. In Ref. [29], 20 million-year-old amber was investigated by a calorimetric and stress relaxation experiment. The authors found that there were no signatures of a diverging timescale below *T_g_*. The analysis of the relaxation time data for 67 glass formers using the universal parabolic law for the activation energy [30] led the authors to the same conclusion.

**Figure 2:**
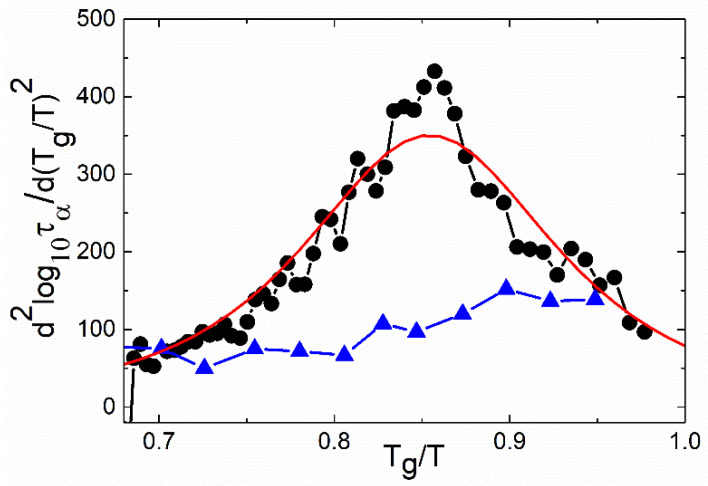
Second derivative of the α relaxation time over *T_g_*/*T* in salicylic acid (salol) (circles) and glycerol (triangles). The data are from Ref. [31]. The solid line is the second derivative of the fit of the relaxation time by the Cohen-Grest Function (14).

Recently, a second derivative analysis of the temperature dependence of structural relaxation was proposed to address this point [31]. If structural relaxation has an Arrhenius temperature dependence, then the second derivative, *d*^2^(ln[*τ_α_*])/*d*^2^(1/*T*), must be equal to zero [31]. This analysis revealed that, indeed, for many systems, the second derivative is close to zero at a high *T*, reflecting the well-known high-temperature Arrhenius behavior, and then go through a maximum and decreases towards zero as *T_g_* is approached, as is shown for salicylic acid (salol) in Figure 2.

This result clearly indicates the presence of low-temperature Arrhenius behavior and suggests no divergence of the time scale at a finite temperature [31]. However, the same analysis revealed no maximum in the second derivative for some other systems (e.g., glycerol) in the temperature range down to *T_g_* (Figure 2). The authors speculated that the maximum might be at temperatures below *T_g_* and is not accessible for this analysis [31].

Therefore, it appears that some systems clearly avoid the divergence of the time scale (as salol in Figure 2), while other systems exhibit a possible divergence all the way down to *T_g_* (as glycerol in Figure 2). Thus, the question of divergence remains at the center of the current discussions [1,25,29,32] and, so far, has no definitive answer. In the following sections, we will review how different models have approached this question.

### 2.2. Free Volume Approach

One of the first and physically appealing approaches to describing the temperature dependence of *η* or *τ_α_* is based on the free-volume ideas [33,34,35,36,37]. This model assumes that the viscosity or relaxation time in liquids can be described by the Doolittle fluidity equation:

*η = η*_0_exp[*v*_0_/*v_f_*] (12)

where *v_f_* is the free volume of the liquid, and *v*_0_ is a constant of the order of the molecular volume. The disadvantage of this model is that the free volume is not a well-defined parameter, especially in the case of covalent- and hydrogen-bonded materials. This model, although it catches some important properties of the glass transition, fails to describe the pressure dependence of the viscosity [38]. The description of the isochoric processes by Equation (12) obviously requires some significant temperature dependence of the parameter *v*_0_, which is hard to account for in the model. The fragility in this model is determined by the temperature derivative of *v_f_,*

*m* = *m*_0_*T*_*g*_*α_f_* (13)

where *α_f_* = *d*ln*v_f_*/*dT* is the temperature expansion coefficient of the free volume. Assuming that the free volume is zero at some temperature *T*_0_, and using linear expansion, one can obtain the VFT equation for *η* (Equation (2)).

A more sophisticated free volume theory by Cohen and Grest predicts, in some approximation, the temperature dependence of *v_f_* and the following expression for *η* [39]:

(14) $$\log\eta /\eta_{0}=\frac{2B}{T-T_{CG}+\sqrt{{\left(T-T_{CG}\right)}^{2}+\alpha T}}$$

where *α* is a constant, and *T_CG_* is a temperature parameter that, in various materials, can be both higher and lower than *T_g_*. In comparison with the VFT function, the Cohen and Grest (CG) equation for *η* has an additional parameter: *α*. The CG function turns into VFT in the limiting case when *α* → 0 and *T_CG_ = T*_0_. The ratio *α*/*T_g_* for various glass formers is rather small, ~0.01 ÷ 0.1 [38]. The function Equation (14) describes *η* and *τ* very well over the entire temperature range, even for the systems in which a single VFT approximation failed [39]. The corresponding fragility is equal to

(15) $$m=\frac{T_{g}m_{0}^{2}}{B}\frac{1+m_{0}\alpha /4B}{1+m_{0}^{2}\alpha T_{g}/4B^{2}}$$

We note that the naive free volume theory predicts the divergence of *τ_α_* at *T*_0_, but the more elaborated free volume theory of Cohen and Grest [39] predicts no divergence of the structural relaxation time. An analysis of the second derivative of the temperature variations of the structural relaxation time reveals that the Cohen and Grest model is the only one that can describe the observed maximum (Figure 2) [31].

### 2.3. Elastic Models

The importance of the mechanical modulus in structural relaxation was discussed by Nemilov [13,40]. A more complex elastic model, the so-called shoving model proposed by Dyre [41,42,43], emphasizes that the act of molecular rearrangement—a barrier crossing or a flow event—takes a very short time in itself. Therefore, during such a process, the environment behaves as an elastic solid, and the infinite frequency shear modulus must be of primary importance for such a barrier crossing. Two types of expressions follow from this theory. The first relates the activation energy of the structural relaxation to the infinite frequency shear modulus *G*_∞_(*T*) [40,43,44]:

(16) $$\log(\tau_{\alpha }\left(T\right)/\tau_{0})=\frac{G_{\infty }\left(T\right)V_{0}}{T}$$

where *V*_0_ is of the order of molecular volume that is assumed to be temperature-independent. Actually, there is no solid justification why *V*_0_ should be temperature-independent. It may change, at least as the inverse density. The shear modulus appears in the activation energy, since the molecular rearrangement leading to the breaking of a cage corresponds to purely shear displacements. With a decrease in the temperature in supercooled liquid, *G*_∞_(*T*) increases, which leads to a super-Arrhenius behavior of viscosity and structural relaxation time. This qualitatively corresponds to the behavior of the activation energy of supercooled liquids.

As discussed in Ref. [45], the shear modulus *G*_∞_ is related to the mean-squared atomic displacement (MSD), 〈*u*^2^〉, on the time scales of the plateau of the intermediate scattering function, i.e., between the average molecular collision time and the structural relaxation time. In particular, for a simple Maxwell model in combination with a Langevin model for Brownian motion, one obtains [46]:

〈*u*^2^(*T*)〉 = 2*T*/*πRG*_∞_ (17)

where *R* denotes the particle radius. Thus, the ratio of the high-frequency elastic constant to the temperature in (16) may be replaced by the inverse MSD. This leads to another relation, proposed by the elastic theory of relaxation in supercooled liquids:

(18) $$\log(\tau_{\alpha }\left(T\right)/\tau_{0})=\frac{\lambda a^{2}}{\langle u^{2}\left(T\right)\rangle }$$

where *a* is the average intermolecular distance, and *λ*~1 is a constant. Such a type of relation was also obtained in Refs. [47,48,49,50,51,52,53]. The fragility in the elastic model is equal to

(19) $$m=m_{0}T_{g}{\left.{\left(\ln\langle u^{2}\rangle \right)}^{\prime }\right|}_{T=T_{g}}$$

or

(20) $$m=m_{0}\left[1-T_{g}\left({\left(\ln G_{\infty }\right)}^{\prime }+{(\ln\;V_{0})}^{\prime }\right)\right){\left.]\right|}_{T=T_{g}}$$

It means that the fragility is defined by the relative temperature variation of 〈*u*^2^〉 or *G*_∞_ and, in principle, *V*_0_.

### 2.4. Entropic Models

One of the most-recognized models for glass transition is the Adam–Gibbs (AG) model [54], which is based on the role of configuration entropy and cooperativity in structural relaxation in liquids. In this model, the activation energy is expressed in terms of the configuration entropy *S_c_*. The AG model assumes that the relaxation events are cooperative and occur in cooperatively rearranging regions (CRR). It is assumed that the number of configurations *N_c_* available for each CRR is constantly independent of the size of the CRR. Then, if the number of molecules in each CRR is *n* and the total number of molecules is *N*, the number of CRRs is *N/n*, and the configuration entropy per molecule is *S_c_* ~ (1/*N*)log *N*_c_*^N/n^* ~ *s_c_*/*n*, where *s_c_* = *k*_*B*_log*N_c_* = *const* is usually equal to *k_B_*ln2. The AG model also assumes that the activation energy of the structural relaxation is proportional to the CRR size *n*; thus, *E_act_* ∝ *n* ∝ 1/*S_c_*. As a result,

(21) $$\log\tau \left(T\right)/\tau_{0}=\frac{B}{TS_{c}\left(T\right)}$$

where *B* = Δ*μs_c_*, is a constant, and Δ*μ* is the energy barrier for individual particle relaxation (no cooperativity). This model describes many experimental (e.g., [55,56,57,58]) and computer simulations (e.g., [59,60,61]) results. Note that some model assumptions, such as that the CRRs are independent and equivalent, the number of accessible configurations is constant, and the activation energy is proportional to the size of the CRR, are not well-justified [62]. Nevertheless, the AG model leads to the thermodynamic expression for fragility:

(22) $$m=m_{0}\left(1+\frac{T_{g}S_{c}^{\prime }\left(T_{g}\right)}{S_{c}\left(T_{g}\right)}\right)$$

where *S_c_*’(*T_g_*) is the temperature derivative of *S_c_*(*T*) at *T_g_*. The AG model assumes that, upon cooling, *S_c_*(*T*) vanishes at some *T_K_*, called the Kauzmann temperature. This scenario explicitly assumes the underlying phase transition at *T_K_*, where the configurational entropy must vanish. It also predicts the behavior for *S_c_*(*T*):

(23) $$S_{c}\left(T\right)=∆c_{p}\ln\left(T/T_{K}\right)\;\approx ∆c_{p}\left(T-T_{K}\right)/T.$$

where Δ*c_p_*(*T*) is the difference of the specific heat between the supercooled liquid and crystalline states. In this approximation, the AG model turns into the VFT function for *τ_α_*(*T*) with *T*_0_ = *T_K_*. Indeed, in many cases, *T*_0_~*T_K_*, but there are supercooled liquids where this rule is significantly violated [63]. With this approximation of *S_c_*(*T*), Equation (22) gives

(24) $$m=\frac{m_{0}}{1-T_{K}/T_{g}}$$

Alternatively, (22) can be presented as a function of the specific heat jump at *T_g_*:

(25) $$m=m_{0}\left(1+\frac{∆c_{p}}{S_{c}\left(T_{g}\right)}\right)$$

A similar relationship between *m* and Δ*c_p_* was derived within the random first-order (RFOT) or mosaic theory of the glass transition [64,65,66,67]. Contrary to the Adam–Gibbs model, the CRR in RFOT can access many configurational states. However, this number is limited by the surface energy needed to compensate for the mismatch between different configurations of the CRR. The surface energy is determined by the surface tension *σ* and the surface area, which, in a simple 3D case, is proportional to *R*^2^. In a more general sense, *σ* is considered as a generalized surface tension, and the surface energy is *σR^θ^*, where *θ* *≤* 2 is an exponent reflecting the complex topography of the surface. The free energy cost to create a CRR of the radius *R* is then similar to the case of conventional nucleation:

Δ*F* = *σR^θ^* − *TS*_*c*_*R*^3^ (26)

The balance of two driving forces determines the characteristic sizes of the rearranging regions ξ:

(27) $$\xi ={\left(\frac{\sigma }{TS_{c}}\right)}^{\frac{1}{3-\theta }}\propto \frac{1}{{\left(T-T_{K}\right)}^{1/\left(3-\theta \right)}}$$

RFOT assumes the free energy barrier scales with the size of the rearranging region as

(28) $$E\propto \xi^{\psi }$$

Qualitatively, this is similar to the Adam–Gibbs relation, *ξ*^3^ ∝ 1/*S_c_*. However, RFOT predicts a stronger dependence of the CRR size on *S_c_*. In particular, if ξ scales are the size of the critical nucleus, where Δ*F* (26) is the maximum, then *ψ = θ*. For *θ* = 2 (2D surface), *ξ* ∝ 1/*S_c_* and *E* ∝ 1/*S_c_*^2^. The theory does not predict the exact values of the exponents *ψ* and *θ*, but they should obey the inequality *θ* ≤ *ψ* ≤ *d* − 1 [68]. The VFT behavior basically corresponds to *θ* = 3/2 case. Actually, this value of the exponent in Equation (28) was obtained in Refs. [64,65,69] on the basis of the renormalization group arguments. The approximation *S_c_*(*T*)~$∆c_{p}\left(T-T_{K}\right)/T$ and the Lindemann criterion of melting provide the VFT behavior [65]:

(29) $$∆F/T=\frac{DT_{k}}{T-T_{k}}$$

with

*D* = 32*R*/Δ*c*_*p*_. (30)

where *R* is the gas constant. The parameter *D* is equivalent to *B/T*_0_ in the VFT function (2) and also characterizes the fragility. It is connected to the usual fragility *m* as

*m = m*_0_ + 2.3 *m*_0_^2^/*D* (31)

The generalized entropy theory of glass formation in polymers (ETGF) [45,70,71] combines the lattice cluster theory (extended to allow for different rigidities of the chain backbone and side groups) with the Adam–Gibbs [54] and Gibbs–DiMarzio [72] theories of glass transition. It predicts systematic changes in the fragility with the chain stiffness, cohesive energy, length of the polymer chain, and of the side chain. One of the important parameters of the theory is the relative rigidity of the chain backbone and the side groups, which strongly affects the fragility. The generalized entropy theory predicts the *τ_α_*(*T*) in the entire temperature range. In particular, at intermediate temperatures, it predicts the scaling, proposed by Rössler [73], log*τ_α_* = *f*(*T_c_*/*T* − 1). As other entropic models, ETGF predicts the divergence of the relaxation time at some finite temperature *T_K_*. The authors actually admit that this result may be an artifact of the mean field approximation [71].

ETGF also reproduces approximate relationship between isothermal compressibility *κ_T_* and configurational entropy *S_c_*, as in the Salzman–Schweizer theory [74,75,76] (will be discussed below). Under the additional assumption that 1/*κ_T_* ∝ *G_∞_*, as argued in Ref. [45], ETGF reproduces the idea of the elastic theory of the relaxation in supercooled liquids with *E_act_ = G_∞_V*. Comparing with the Adam–Gibbs expression (Equation (21)), the authors of Ref. [45] obtained the relation between configuration entropy and MSD: *S_c_T* ∝ 〈*u*^2^〉. The same scaling for landscape configurational entropy and MSD was suggested as a result of molecular dynamic simulations [77]. Thus, EGTF attempts to reveal a possible connection between entropic and elastic models of the relaxation in supercooled liquids.

To reveal this connection, Wyart [78] argued that if the relationship between relaxation time and MSD (18) exists, then there is a relationship between fragility and vibrational entropy, which leads to *m* = 52Δ*C_vib_*/3*α*, where *α* is a numerical parameter, which reflects the frequency dependence of the anharmonicity. An increase in 〈*u*^2^〉 due to the softening of low-frequency vibrational modes (boson peak, see description below) can significantly affect the temperature dependence of *τ_α_*. This idea was further developed [79] for materials with a rigidity transition, and a possible connection of fragility to the elastic properties of the liquid and to the boson peak was revealed.

### 2.5. Other Model Approaches

Salzman and Schweizer [74,75] combined the methods of the mode coupling, density functional, and activated hopping transport theories to extend the mode-coupling theory to the activated hopping regime in the temperature range down to *T_g_*. They used coarse graining and condensed all the specific materials’ chemistry to a few parameters, such as dimensionless compressibility *S*_0_ and packing *p*. An important parameter of the theory is the MCT-like critical temperature *T_c_*, below which activated hopping begins to affect the dynamics. Following the empirical findings [49], the theory assumes that, at *T_c_*, the segmental relaxation time has a universal value of ~10^−7^ s. Salzman and Schweizer show that log*τ_α_*(*T*) scales are a universal function of [(*T_c_/T* − 1)/(*T_c_/T_g_* − 1)]^Δ^, the scaling previously proposed by Rössler et al. [73] based on experimental studies. The origin of the scaling behavior is the critical power–law growth of the collective barrier. The dynamic fragility in this theory can be expressed in terms of the *T_c_/T_g_* ratio:

(32) $$m=\frac{b}{1-T_{g}/T_{c}}$$

where *b* ≈ 14 ± 2 for *τ*(*T*_c_) = 10^−7±1^ s and *τ*(*T_g_*) = 100 s. A similar result was obtained in the model of percolation of solid-like clusters [80]. The density, the entropic contribution to the dimensionless compressibility *A*, and the temperature independent cooperativity factor *a_c_* are critical parameters in the Salzman and Schweizer theory that determine the *T_c_*/*T_g_* ratio and fragility. The cooperativity factor *a_c_* is not a priori predicted by the theory but has the meaning of the number of dynamically correlated segments along the chain and is usually assumed to be *a_c_* = 2 ÷ 5. In this respect, fragilities 60–70 correspond to a small collective factor *a_c_*~1, i.e., flexible polymers, while polymers with stiff backbones, bulky side groups, or polar monomers have large *a_c_* and fragilities *m*~100–180. However, the theory is a single particle mean field approximation. Collective effects cannot be estimated within this version of the theory.

Mirigian and Schweizer explicitly took into account the collective effects in the next improved version of the theory [81,82,83]. They included the collective effects associated with long-range elastic distortion of the liquid into the elastically collective nonlinear Langevin equation (ECNLE) theory. The theory is applied to hard sphere fluids [82] and thermal liquids [83]. The chemical complexity of the thermal liquids is taken into account by nanometer scale coarse graining and mapping to a hard sphere fluid with some packing fraction, which reproduces the experimental dimensionless compressibility *S*_0_ of the specific system, *S*(*k*→0) ≡ *S*_0_ = *ρk_B_Tκ_T_* ∝〈(*δρ*)^2^〉, where *S*(*k*) is the static structure factor, *κ_T_* is the compressibility, and *ρ* is particle density [83]. In the case of polymers, the ECNLE theory uses the same mapping approach at the Kuhn segment scale [84,85,86]. The theory predicts that, in the high-temperature regime, when the collective effects are negligible, log(*τ_α_*/*τ*_0_) ∝ *S*_0_^−1^. At low temperatures, where the collective effects due to cooperative elastic distortions that arise in the course of cage breaking become significant, log(*τ_α_*/*τ*_0_) ∝ *S*_0_^−3^. The theory identifies *S*_0_ as the key ‘thermodynamic order parameter’ for all the activated regimes. The latter scaling includes both the cage and elastic barrier contributions. In this regime, the two barriers exhibit a nontrivial ‘compensation effect’ [87] in their contribution to the total barrier, resulting in a predicted cubic power law. Additionally, in this deeply supercooled regime, the equivalent representation is log(*τ_α_*/*τ*_0_) ∝ *σ*^3^*G*/*T*, where σ is the molecular size, and *G* is the dynamically relaxed high-frequency elastic shear modulus. This result is similar to the prediction of the shoving model [43], but here, it arises on the basis of a different mechanism. In Refs. [86,88], the predictions of the theory are compared to the experimental results for *α*-relaxation time in glass formers of different chemical compositions, fragility, and in different temperature ranges. It is shown that the high-temperature renormalized Arrhenius scaling of log(*τ_α_*/*τ*_0_) vs. *S*_0_^−1^ applies over ~1.5–3 decades, roughly double or more the range that classic Arrhenius behavior applies. The interval is longer in less fragile systems, e.g., in glycerol in comparison with salol and toluene. At low temperatures, *α*-relaxation time scales with *S*_0_^−3^, and the interval of the scaling is longer in more fragile systems, reaching, e.g., ~14 decades in toluene, ~15 in sorbitol, and about 7 decades in glycerol. A master curve was constructed as a superposition of the high- and low-temperature regimes with the help of an adjustable dimensionless weight parameter w that captures the noncooperative-to-cooperative crossover:

log(*τ_α_/τ*_0_) = *w*(*S*_0_(*T_g_*)/*S*_0_(*T*)) +(1 − *w*) (*S*_0_(*T_g_*)/*S*_0_(*T*))^3^ (33)

The authors found that the parameter *w* correlates very well with the fragility, ln (1/*w*) = 0.0493(*m* − 42.48) [88]. Since *w* ≥ 0, it can be concluded that the collective elasticity effects are irrelevant for materials with fragility less than 41.5, such as boron oxide and silica.

Another approach describing glass transition is the frustration-limited domain theory [89,90]. This theory assumes the existence of a locally preferred structure that is different from the structure of the respective crystal. The theory is based on the presence of frustration in the structures of liquids. This means that the preferable local order cannot be extended over long distances, and thus, frustration appears on some mesoscopic length scale, and a mosaic pattern of domains occurs. The domains are separated by interfaces with a higher concentration of defects. Cooperative dynamics in the domains leads to a super-Arrhenius temperature dependence of the structural relaxation at sufficiently low temperatures. This theory predicts that fragility depends on frustration, because larger frustration means a smaller collectively relaxing domain size that corresponds to a lower fragility.

Scaling arguments lead to the following expression for the activation energy in the frustration-limited domain theory: *E*(*T*) = *E*_∞_ + Δ*E*(*T*), where:

(34) $$∆E\left(T\right)=\left\{\begin{array}{c} 0,\;\;\;\;T>T_{A}\\ BT_{A}{\left(1-T/T_{A}\right)}^{y} \end{array}\right.$$

Here, *T_A_*, *y*, and *B* are fitting parameters that provide a good description of the experimental data. *T_A_* is higher than the melting temperature *T_m_* and corresponds to the temperature above which the Arrhenius regime dominates. The exponent *y* was obtained in the range 7/3 ÷ 3 for different materials with a typical value of 8/3. The factor *B* describes the degree of frustration—the higher frustration, the lower the *B*. The fragility, estimated with the activation energy (34) is equal to

*m* = *m*_0_ + *By*(1 − *T_g_*/*T_A_*)*^y^* (35)

Thus, increasing the frustration in the molecular arrangements leads to decreasing the fragility.

Another theory of the glass transition is based on the ideas of dynamical facilitation [91]. In simple words, the latter takes into account the fact that the elementary structural relaxation events in supercooled liquids are comparatively reared on a molecular time scale, and in a local region where such event occurs, the probability of the relaxation of nearby molecules increases. In this approach, it is not geometry but, rather, local mobility that is central for the theory. There are many realizations of the dynamical facilitation scenario in various models [91,92], where the local kinetics are constrained by various conditions. Elmatad, Chandler, and Garrahan [30] obtained a very simple and universal expression for the structural relaxation time in a class of kinetically constrained models:

log(*τ*/*τ*_0_) = (*J*/*T*_0_)^2^(*T*_0_/*T* − 1)^2^, *T*_0_ > *T* > *T_x_* (36)

The temperatures *T*_0_ and *T_x_* determine the applicability of Formula (36). *T*_0_ is a high temperature at which the concentration *c* of excitations that facilitate the local mobility is so high that no correlated motion is required. It has the meaning of the onset of glassy dynamics. The temperature *T_x_* is low enough that the super-Arrhenius rate related to the constrains becomes so small that the molecule would prefer to avoid the constrains, although at some energy cost. Therefore, below *T_x_*, *τ_α_* will eventually return to the Arrhenius dependence. The predicted parabolic law (Equation (36)) is explained by the logarithmic dependence of the activation energy *E* for relaxation of the domain on its size ξ. The latter depends on *c*, e.g., *ξ*^3^ ∝ *c* in three dimensions. Since *c* is described by the Boltzmann distribution, log*c* ∝ log*ξ* ∝ *E* ∝ 1/*T*. It has been shown that the three-parameter parabolic Equation (36) describes well the experimental data in 67 glass formers with high and intermediate fragility down to *T*_g_ [30]. This model has also been tested in extensive molecular dynamic simulations of five distinct simple liquid mixtures [93]. The fragility in this model is basically determined by the ratio *J*/*T*_0_, where the parameter *J* has the meaning of the energy scale for the excitations of the correlated dynamics:

*m* = 2(*J*/*T_g_*)^2^(1 − *T_g_*/*T*_0_) (37)

Since Equation (36) is not applicable at sufficiently high *T*, it is impossible to put log(*τ*(*T_g_*)/*τ*_0_) equal to *m*_0_ and, thus, reduce the number of parameters in Equation (37), as it was, e.g., in the VFT case.

We want to emphasize the essential difference between this model and the Adam–Gibbs approach. The latter assumes domains of collective relaxations, CRR, that increase in size upon cooling, while, in the dynamical facilitation picture, the main object is the elementary excitations with temperature-independent sizes. With the decreasing *T*, their concentration decreases, and the distance between the excitations increases. This leads to an increase in the dynamical heterogeneity length scale. It is not clear whether both pictures may be connected.

## 3. Experimental Tests of Different Models: Search for Cooperativity

### 3.1. Thermodynamic Properties

**Figure 3:**
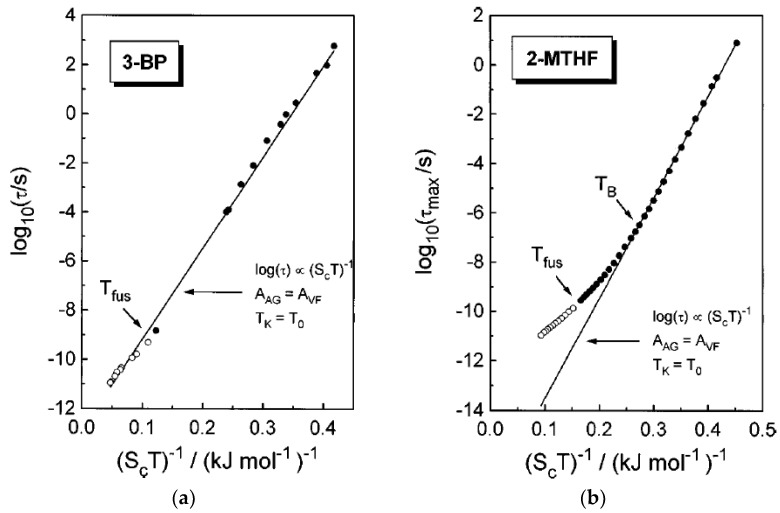
Adam–Gibbs plot log*τ_α_* vs. 1/*S_c_T* for 3-bromopentane (**a**) (3-BP) and 2-methyltetrahydrofuran (**b**) (2-MTHF). Deviations from the AG prediction at high temperatures is obvious for 2-MTHF. Reprinted from [56] with the permission of AIP Publishing.

According to the AG model, the relaxation time is controlled by a variation of configurational entropy, log*τ_α_* ∝ 1/*S_c_* [54]. This suggests a direct experimental test of the model prediction through comparison of the relaxation time and thermodynamic measurements. However, this test is not straightforward, because it is not obvious what should be taken as the configurational entropy *S_c_*. Measurements of specific heat Δ*c_p_* give a result that corresponds to integration over all degrees of freedom, including vibrational and intramolecular. As shown in Ref. [94], the configuration entropy may have contributions that are irrelevant for the glass transition (e.g., related to secondary relaxations) and so should not be taken into account in the AG model. In most cases, the configuration entropy is taken as the difference between entropy of the supercooled liquid and corresponding crystal, *S_c_*(*T*) = *S_liq_*(*T*) − *S_cryst_*(*T*) [54,55,56,57]. Indeed, this approach finds a good correlation between temperature variations of the relaxation time and so-defined *S_c_*(*T*) in many glass-forming liquids (see, e.g., Figure 3) at low *T*. However, depending on the fragility, deviations can occur at higher *T*, usually at *T > T_B_*_,_ where *T_B_* is the so-called Stickel temperature close to the MCT *T_c_*. A good correlation of logτ and 1/*S_c_* has also been found in simulations [59,60,61]. All these studies essentially confirm that, for many materials, the Kauzmann temperature *T_K_* is indeed close to the VFT temperature *T*_0_.

**Figure 4:**
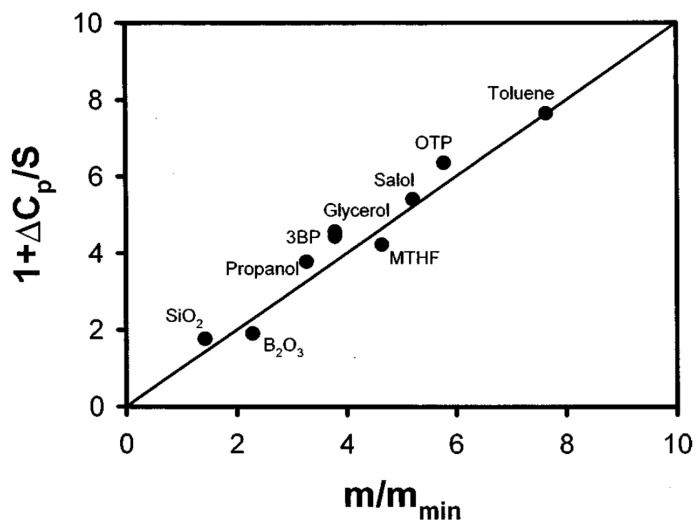
Experimental verification of the predicted relationship (25) between the thermodynamic and kinetic parameters for materials spanning a wide range of fragilities. The solid line is a guide for the eye, and *m_min_* ≡ *m*_0_ = 17. Reprinted from [58] with the permission of AIP Publishing.

A direct comparison of the predicted by AG model relationship between thermodynamic and kinetic fragilities (25) was performed in [58] for various glass formers. A very good correlation (Figure 4) provides strong support for the AG approach.

**Figure 5:**
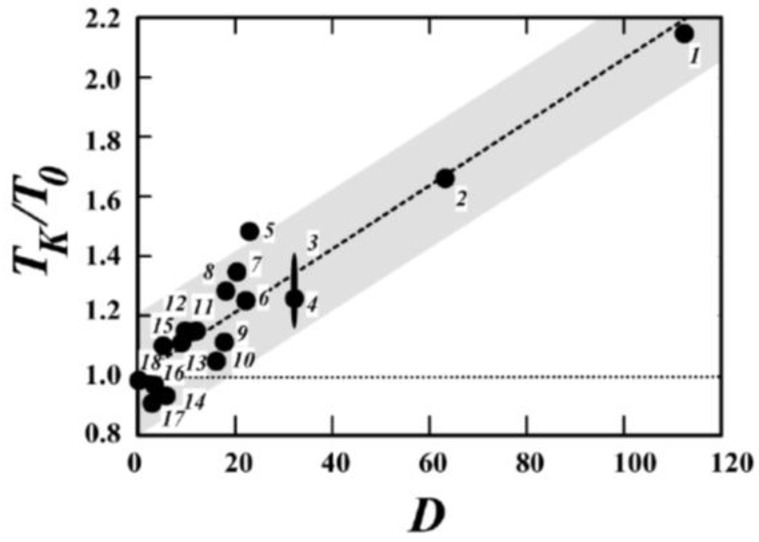
Correlation between *T_K_*/*T*_0_ and the VFT parameter *D* related to the fragility index through Equation (31). Reprinted figure with permission from [63]. Copyright (2003) by the American Physical Society.

However, detailed studies on a wider range of materials, including polymers, revealed that the proposed ratio between log*τ* and 1/*S_c_* is violated in many systems [62]. Moreover, *T_K_* and *T*_0_ differ strongly for these materials, and according to [63], the ratio *T_K_*/*T*_0_ actually seems to increase with the decreasing fragility (Figure 5). The authors used the VFT parameter *D* = *B*/*T*_0_ = 2.3 *m*_0_^2^/(*m* − *m*_0_) as another measure of the fragility defined by Equation (31).

The RFOT theory predicts a more general expression for the relationship between the relaxation time and configurational entropy (Equation (28)), log*τ_α_* ∝ 1/(*TS_c_*)*^ψθ^*^/(3−*θ*)^. In this case, by adjusting the exponents *ψ* and *θ*, one can reach a better agreement between the temperature variations of the structural relaxation time and entropy [64,65]. However, computer simulations and experiments give values of *θ* = 2 ÷ 2.3 and *ψ* < 1 [64], which are difficult to explain within RFOT. Another explanation could be a contribution of additional processes to the total configuration entropy that should be subtracted [94,95,96]. Thus, it remains unclear whether the failure of the AG relationship (Equation (21)) is a consequence of additional contributions to the measured entropy or due to the more complex relationship between ln*τ_α_* and 1/*S_c_* (as, e.g., proposed in RFOT).

**Figure 6:**
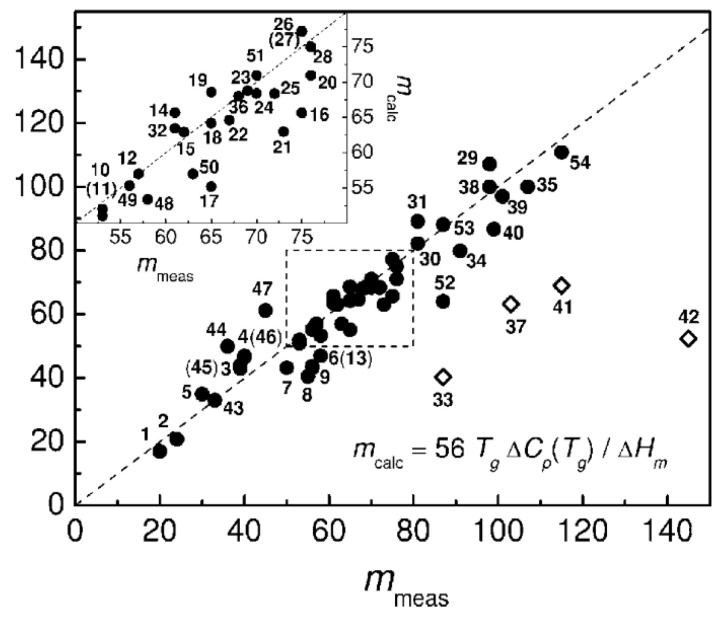
Correlation of the calculated using Equation (38) thermodynamic fragility *m_calc_* with the measured *m_meas_* fragility index for 54 nonpolymeric glass-forming materials. The data in the 50 < *m* < 80 range are also shown in the enlarged inset for clarity. The dashed line represents the relation of Equation (38). Reprinted from [99] with the permission of AIP Publishing.

Thermodynamic models also suggest that the jump in specific heat Δ*c_p_* at *T_g_* due to the freezing of configurational degrees of freedom should increase with *m*. The relationship between the kinetic fragility *m* and the thermodynamic properties of the supercooled liquids has been discussed in many papers [56,67,97]. Based on an analysis of the many glass-forming systems, Wang and Angell [98,99] suggested a simple empirical relation between Δ*c_p_* and *m* (Figure 6):

(38) $$m=56\frac{T_{g}∆c_{p}}{∆H_{m}}$$

where Δ*H_m_* is the melting enthalpy. A similar expression with a slightly different pre-factor was derived within RFOT [66,67]. However, not all systems follow this rule, and strong deviations from the expected correlations are found for some molecular liquids (Figure 6). As discussed in Ref. [99], the materials that deviate strongly from the proposed relationship, selenium (33), toluene (37), triphenylphosphite (TPP) (41), and decalin (42), have special reasons for the deviation. Selenium has a temperature-dependent fraction of rings, TPP has a glacial phase, decalin is a 50–50 mixture of the cis- and trans-isomers, while toluene has unusually high fragility for materials of this class. The polymers also deviate from this rule and will be discussed separately in Section 5.

Using thermodynamic arguments, the authors of Ref. [99] showed that there is an upper limit for fragility. For the molecular systems, they obtained the upper limit of about 173. This value is also supported for nonpolymeric systems by the correlation between fragility and the stretching exponent *β_KWW_* (Kohlrausch-Williams-Watts, KWW) [5]. In Ref. [100], the enthalpy relaxation measurements during cooling and heating across the glass transition were used to determine *m_max_*. The extrapolation of the relaxation enthalpy to zero predicted the upper limit of fragility in the interval ~170–180. The value *m_max_*~175–180 follows also from the ratio of the relaxation widths of structural *α*- and *β*-relaxations [100,101] and from the ratio of the configurational heat capacity and the total heat capacity jump at the glass transition [100,102].

### 3.2. Role of Cooperativity/Heterogeneity

The key in the entropic models is the assumption of the increase in cooperativity of structural relaxation upon approaching *T_g_*. These models implicitly relate higher fragility to higher molecular cooperativity at *T_g_* [54]. Attempts to experimentally test this relationship remain at the center of many studies. The largest challenge here is how to measure cooperativity in structural relaxation. There is no accepted definition and no direct experimental measure of the cooperativity. In many cases, one can measure the length scale of dynamic heterogeneity *ξ*, and then, it is assumed that *ξ* reflects the length scale of cooperativity. Although the analysis of MD simulations suggested some ways to differentiate the cooperativity and heterogeneity length scale [103], no consensus on this topic has been achieved so far. This remains one of the important questions in the field: How can we quantify the cooperativity, and how is it related to the length scale of dynamic heterogeneity?

Several ideas have been proposed for measurements of the length scale of dynamic heterogeneity. First of all, it has been shown that the experimentally measured ensemble averaged two-point correlation functions (e.g., neutron or light scattering intensity, dielectric permittivity, etc.) cannot provide information on the dynamic heterogeneity, and measurements of higher order correlation functions are required [104,105]. Detailed discussion of this problem and of the recent results on this topic are presented in the book [105]. It is fairly easy to analyze the higher-order correlation functions in simulations where knowledge of the trajectories for all the atoms at any time provides a possibility to calculate any correlation functions. The traditional focus is on four-point correlation functions [106] that measure the extent of dynamical heterogeneity, i.e., how the mobility of different particles in the media are correlated. Clearly, the mobility itself depends on the correlation of density at different points at different times, i.e., is a two-point correlation function. If the respective mobility for the *i*-particle is *c_i_*(*t*,0), then the mobility field is introduced as [106].

(39) $$c\left(r;t,0\right)=\sum_{i}c_{i}\left(t,0\right)\delta \left(r-r_{i}\right)$$

The four-point correlation function is defined through the spatial correlation of the mobility field:

(40) $$\;G_{4}\left(r;t\right)=\langle c\left(r;t,0\right)c\left(0;t,0\right)\rangle -\langle c{\left(0;t,0\right)\rangle }^{2}$$

The time-dependent four-point susceptibility is

(41) $$\;\chi_{4}\left(t\right)=\int \;G_{4}\left(r;t\right)dr$$

*χ*_4_(*t*) can be expressed via the fluctuation of density *δρ*(***r****,t*) = *ρ*(***r****,t*) − *ρ*_0_, where *ρ*_0_ is the average density [107]:

(42) $$\chi_{4}\left(t\right)=\int dr_{1}dr_{2}[\langle \delta \varrho \left(r_{1},0\right)\delta \varrho \left(r_{2},0\right)\delta \varrho \left(r_{1},t\right)\delta \varrho \left(r_{2},t\right)\rangle -\;\;\;\;\;\;\;\;\langle \delta \varrho \left(r_{1},0\right)\delta \varrho \left(r_{1},t\right)\rangle \langle \delta \varrho \left(r_{2},0\right)\delta \varrho \left(r_{2},t\right)\rangle ]$$

Assuming a compact structure of the heterogeneities [106],

(43) $$\;\chi_{4}\left(t\right)\propto N_{corr}$$

where *N_corr_* is the number of particles in the dynamically correlated region. The first experimental attempts to measure the length of the dynamical heterogeneity were done with multidimensional NMR, where a particular sequence of pulses was designed to select a sub-ensemble of either the slowest or fastest relaxing units and watch their relaxation behaviors over time [108,109]. These studies clearly demonstrated the presence of dynamic heterogeneities in glass-forming liquids and also revealed that their lifetime is comparable to the average structural relaxation time [108,109]. In other words, the fastest relaxing regions might become the slowest and vice versa on the time scale ~(2 ÷ 3)*τ_α_* [108,109]. Four-dimensional NMR has been used to estimate the length scale *ξ* of dynamic heterogeneities in several materials [108,109]. It appears to be in the range *ξ*~1.5–3 nm, but the accuracy of these estimates was not sufficient to provide any conclusions about their temperature variations or correlation to fragility.

**Figure 7:**
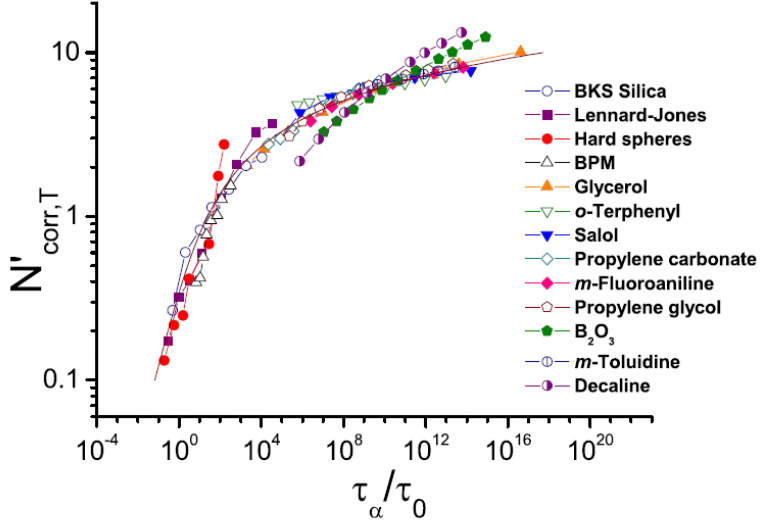
*N_corr_*(*T*) for different materials on a logarithmic scale as a function of *τ_α_*/*τ*_0_. The full line corresponds to the dependence *τ_α_* ∝ *A*(*N_corr_/N*_0_)^γ^exp(*N_corr_/N*_0_)*^ψ^*, with *A* = 4, *N*_0_ = 0.8, γ = 2, and *ψ* = 1.4 that describes a crossover from a power law scaling at high temperatures (small *τ_α_*) to a logarithmic growth close to the glass transition. Using the freedom left by unknown normalizations of order unity, the data are shifted to obtain a better collapse onto the fit. Reprinted figure with permission from [111]. Copyright (2007) by the American Physical Society.

A detailed theoretical analysis suggested that the lower limit of *χ*_4_ can be estimated based on the analysis of the temperature variations of the two-point correlation function *χ_T_* [110,111]. The latter can provide estimates of the number of dynamically correlated particles *N_corr_*. This approach has opened the way for the analysis of *χ_T_* in many materials and has revealed that there is no divergence of the length scale upon cooling, which is expected in the case of the AG model. In contrast, *N_corr_* increases as a logarithmic law upon approaching *T_g_* [111], and there is no correlation of *N_corr_*(*T_g_*) and fragility [110,112]. Moreover, even temperature variations of *N_corr_*(T) appear rather similar for strong and fragile systems, as well as for classical computer simulation models (Figure 7). According to these studies, the correlation length *ξ* changes by only ~20–30% when the liquid is cooled from *T_m_* down to *T_g_* (Figure 7) [111].

**Figure 8:**
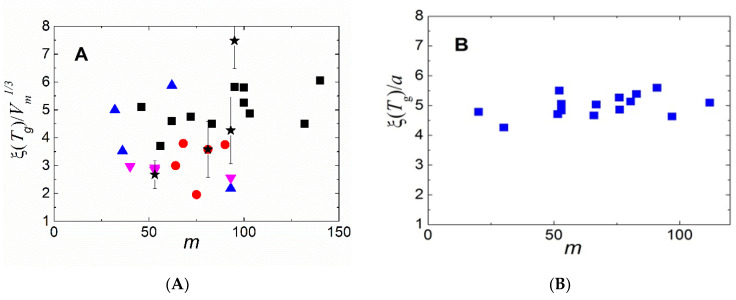
(**A**) ξ/*V_m_*^1/3^ vs. *m* for various glass-forming materials; here, *V_m_* is the molecular volume. ξ is estimated using the boson peak frequency. The materials are covalent and ionic (blue triangles), molecular (red circles), hydrogen-bonding (magenta triangles), and polymeric glasses (black squares). Additionally, *ξ* estimated from the 4D NMR are shown (stars) [112,113]. (**B**) Correlation length *ξ*(*T_g_*) in supercooled liquids, estimated from *χ_T_* at the glass transition expressed in bead units *a.* From [110]. Reprinted with permission from AAAS.

An indirect measurement of the dynamic heterogeneity length scale was suggested based on the analysis of the boson peak spectra [112,113]. The boson peak presents an excess in the vibrational density of states over that expected in the Debye model at low energies (~1 ÷ 5 meV) [114,115]. Although the nature of these excess vibrations remains a topic of active debate [116,117,118], many researchers associate them with fluctuations of elastic constants in a disordered structure [116,117,119,120,121]. In that case, the frequency of the boson peak *ν_BP_* ∝ 1/*ξ_BP_*, where *ξ_BP_* is the correlation length of elastic constant fluctuations [119,120,121]. The analysis of *ξ_BP_* at *T_g_* revealed a good agreement with earlier 4D NMR studies (Figure 8A), providing justification for this method. A recent analysis of the data for many materials [122] confirmed the correlation of *χ_T_* and *ξ_BP_* at *T_g_*. An analysis of a large number of glass-forming systems revealed that there was no correlation between *ξ_BP_*(*T_g_*) and fragility (Figure 8A). Thus, the analysis of multidimensional NMR, *N_corr_*, estimated from *χ_T_* and *ξ_BP_*, did not reveal a direct correlation of the characteristic dynamic heterogeneity length scale at *T_g_* to the fragility of glass-forming liquids (Figure 8).

Recently, several attempts have been made to employ nonlinear dielectric spectroscopy to measure the higher-order correlation function *χ*_3_(*T*) and, in this way, to analyze the dynamic heterogeneity length scale [123,124,125,126,127,128,129,130,131]. It has been shown that the measured *χ*_3_ agrees well with the temperature variations of *N_corr_* estimated from *χ_T_* [123,124,125,126,127,128,129,130,131]. These studies, applied to several glass-forming liquids, indeed found a correlation between the temperature variations of *χ*_3_(*T*) and fragility [127]. However, the entire approach of nonlinear dielectric spectroscopy to the analysis of dynamic heterogeneities was questioned in a recent paper [132].

It has been also suggested that an analysis of the wave vector *Q* dependence of the structural relaxation might reveal the information on the dynamic heterogeneity length scale [133]. Although the scattering spectra are two-point correlation functions, they might have different behaviors when the probe length scale is much larger than *ξ* and much smaller than *ξ*. Thus, some crossover between these two regimes can be expected at *Q*~2*π/ξ*~1.5–5 nm^−1^. However, no systematic studies using this approach has been performed so far.

**Figure 9:**
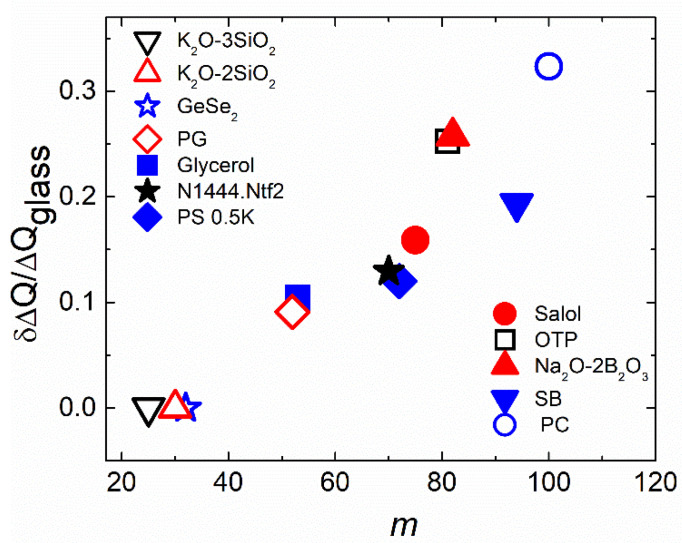
The relative change in the width of the main diffraction peak between *T* = 0.9*T_g_* and *T* = 1.3*T_g_* vs. fragility for several glass-forming systems. Abbreviations: PS—polystyrene, salol—phenyl salicylate, OTP—orthoterphenyl, PG—propylene glycol, SB—sucrose benzoate, PC—propylene carbonate, and N1444.NTf2—room temperature ionic liquid. Data from Ref. [134].

An interesting analysis revealed a possible correlation between the temperature variations of the static properties of liquids to their fragility [134]. The width of the first sharp diffraction peak in static structure factor *S*(*Q*), Δ*Q*, is often considered as a measure of the static correlation length *l_c_* = 2*π*/Δ*Q*, i.e., a kind of measure for the length of the intermediate range order in liquids and glasses. A detailed analysis of *S*(*Q*) in several nonpolymeric glass-forming systems revealed that, in strong systems, Δ*Q* does not show any significant temperature variations, while it narrows significantly upon cooling in fragile glass formers (Figure 9).

**Figure 10:**
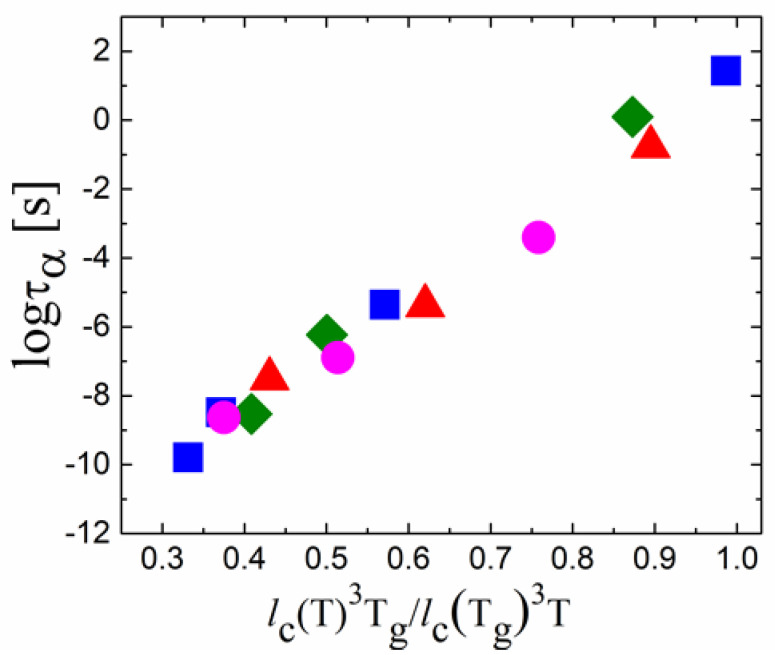
The dependence of log *τ_α_*(*T*) on *l_c_*(*T*)^3^/*T* normalized to its value at *T_g_* for polystyrene (triangles), sucrose benzoate (squares), propylene carbonate (circles), and glycerol (diamond). Data from Ref. [134].

Moreover, it appears that, for many systems, the temperature variations of structural relaxation time follow the variations of *l_c_*^3^(*T*) ∝ Δ*Q*(*T*)^−3^ (Figure 10), as expected in the Adam–Gibbs theory [54] if *l_c_*^3^ changes with *T* as the volume of cooperatively rearranging regions. These results suggest that the change in the static structural order in liquids during cooling can play a significant role in controlling the activation energy of structural relaxation [134].

### 3.3. Role of Density (Free Volume) and Thermal Energy

In contrast to the entropy models, the free volume approach assumes that the primary parameter controlling the change in the relaxation time is the change in density upon cooling [34,39]. In order to separate the role of density and thermal energy in slowing down the structural relaxation, several groups performed detailed studies of the relaxation time as a function of the temperature and pressure (density). A comprehensive review of this topic is presented in [135,136,137], and we will discuss it only briefly, in particular, the scaling of relaxation time on temperature and density proposed in [138].

First of all, one can define isochoric fragility *m_V_*, where the temperature variation of the relaxation time is defined at a constant volume by applying pressure to compensate for the temperature variation of the density [135,136,137]:

(44) $$m_{V}={\left.\frac{\partial \log\tau }{\partial \left(T_{g}/T\right)}\right]}_{V=const,\;T=Tg}$$

**Figure 11:**
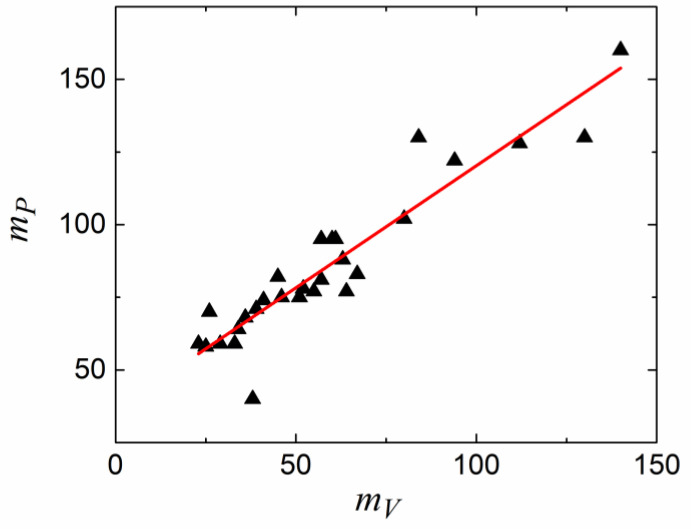
The isobaric fragility vs. the isochoric fragility. Fitting (solid line) gives *m_P_* = (37 ± 3) + (0.84 ± 0.05) *m_V_*. The data is from Ref. [135].

It has been demonstrated that, for most glass-forming liquids, *m_V_* correlates to isobaric (usual) fragility: *m* ≈ 37 ± 0.84 *m_V_* (Figure 11). This relationship clearly fails for liquids with *m* < 37 (i.e., most of the covalent bonding systems). Moreover, H-bonding liquids (e.g., glycerol) deviate strongly from this rule because of their very weak dependence of the relaxation time on pressure [139]. It has been proposed that the relative importance of thermal energy vs. density contributions to the slowing down of structural relaxation can be characterized by the ratio of the apparent activation energy at a constant volume, *E_V_* = [*d*ln(*τ*)/*d*(1/*T*)]*_V_*_=*const*_, to that at a constant pressure, *E_P_* = [*d*ln(*τ*)/*d*(1/*T*)]*_P_*_=*const*_, which is also equal to *m_V_*/*m*. The ratio *E_V_*/*E_P_* should be close to 1 if the energetic contribution dominates and should be lower than 0.5 if the density dominates. An analysis of the experimental data shows that *E_V_*/*E_P_* varies from ~0.9–0.94 in H-bonding systems to ~0.4 in many Van der Waals liquids [135].

The sensitivity of the relaxation time to the pressure is traditionally characterized by the activation volume *ΔV*^#^ [135], defined as:

(45) $$\tau \left(P,T\right)=\tau \left(0,T\right)\exp\left(\frac{P∆V^{\#}}{kT}\right)$$

**Figure 12:**
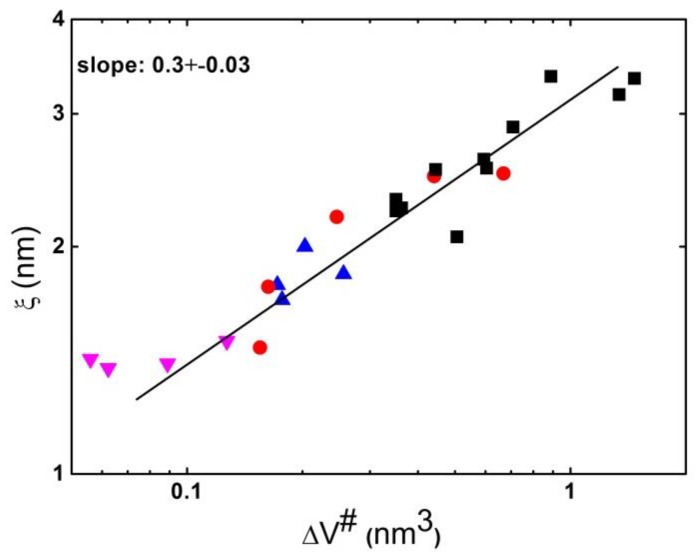
Correlation of *ξ_BP_* and the activation volume. The slope of the log–log plot is ~0.3, suggesting Δ*V^#^∝ξ_BP_*^3^, i.e., the activation volume is a fraction of the heterogeneity volume [112,140]. The materials are covalent and ionic (blue triangles), VdW molecular (red circles), hydrogen-bonding (magenta triangles), and polymeric glasses (black squares).

H-bonding systems usually have a very small activation volume, while polymers have the largest Δ*V*^#^ [135]. The activation volume increases with the cooling [135], reflecting an increase in the dynamics sensitivity to density upon approaching *T_g_*. It is interesting to note that studies performed in [112,113] revealed a good correlation between *ξ_BP_* and the activation volume Δ*V*^#^ at T_g_ (Figure 12). Although some ideas have been recently proposed in [83], this correlation remains a puzzle and might reflect a connection of the pressure (density) sensitivity of structural relaxation to the dynamic heterogeneity length scale [140].

**Figure 13:**
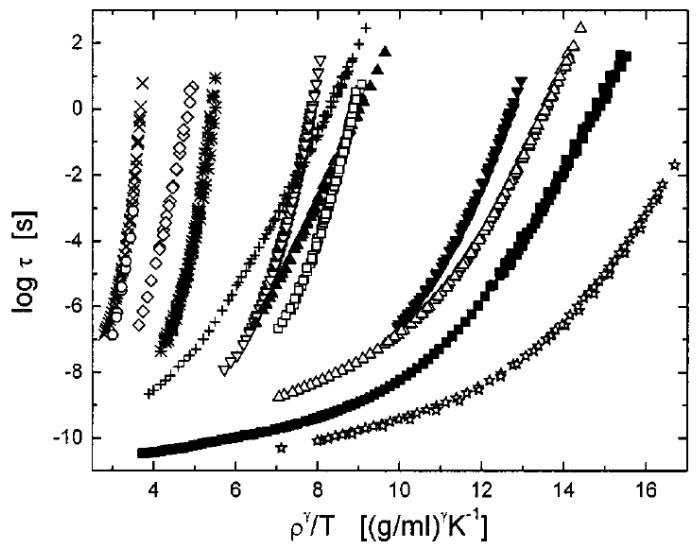
Thermodynamic scaling reflecting the dependence of the relaxation time on the temperature and density in a single plot. It presents the *α*-relaxation times of molecular liquids as a function of the reciprocal of the temperature times the volume in power *γ* [138]. Dielectric relaxation times as a function of the product of the reciprocal temperature and density, the latter raised to the power of *γ =* 1.9 (1,2-polybutadiene ×), 3.0 (1,4-polyisoprene ○), 6.2 (OTP/OPP ◊), 2.5 (poly-propylene glycol ∗), 8.5 (BMMPC +), 3.5 (poly[(phenyl glycidyl ether)-co formaldehyde] *σ*), 7.0 (BMPC *π*), 5.6 (PMPS ☐), 5.0 (PMTS *θ*), 4.5 (phenylphthalein-dimethylether △), 5.2 (salol ■), and 3.7 (propylene carbonate ✯). Reprinted from [138] with the permission of AIP Publishing.

**Figure 14:**
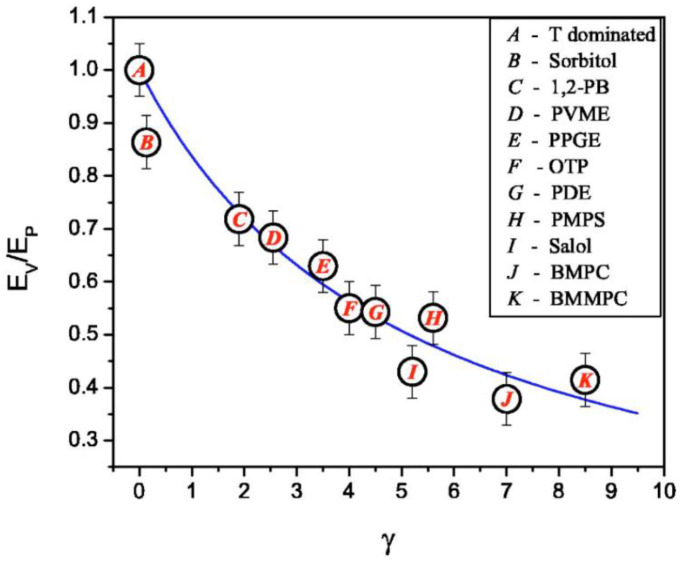
Ratio of isochoric and isobaric activation enthalpies vs. exponent *γ* of the thermodynamic scaling of *τ_α_*(*T*,*P*) [141]. The solid line is the best fit to the data of Equation (47). *B*, D-sorbitol; *C*, 1,2-polybutadiene; *D*, poly(vinyl methyl ether); *E*, poly(phenyl glycidyl ether)-coformaldehyde; *F*, ortho-terphenyl; *G*, phenolphthalein-dimethylether; *H*, polymethylphenylsiloxane; *I*, phenyl salicylate; *J*, 1,1′-bis(p-methoxyphenyl)cyclohexane; and *K*, 1,1′-di(4-methoxy-5-methylphenyl)cyclohexane. Reprinted figure with permission from [141]. Copyright (2004) by the American Physical Society.

The pressure measurements have revealed that the relaxation time for most of the glass-forming liquids follows a universal thermodynamic scaling ln*τ*_α_ ∝ *TV^γ^* (Figure 13) [139,141,142,143]. The exponent *γ* depends on the material and varies from ~0.13 in sorbitol to ~8 in systems such as 1,10-di(4-methoxy-5-methyl phenyl) cyclohexane (BMMPC) [135]. There were several attempts to relate it to the specifics of the intermolecular potential [144,145,146]. It was argued that *γ* is determined by the steepness of the repulsive part of the potential, and in the case of the power–law potential, it corresponds to *U*(*r*) ∝ *r*^−3*γ*^. The interactions with strong directional bonding are much less sensitive to pressure. In particular, hydrogen-bonding materials have a small sensitivity to pressure and, respectively, small *γ* ≤ 1, and they do not even always scale. Exponent γ is also connected to the Grüneisen parameter *γ_G_, γ* = 2*γ_G_* − 2/3, although the exact relation is model-dependent [147]. In Ref. [148], *γ* was expressed via the parameters that can be measured at ambient pressure:

(46) $$\gamma =V∆\alpha_{P}/\left(∆c_{P}\kappa_{T}-TV\alpha_{P}∆\alpha_{P}\right)$$

where *α_P_* denotes the isobaric thermal expansion coefficient, *c_P_* is the specific heat, *κ_T_* is the isothermal compressibility, and Δ denotes the change at the glass transition. This relation was confirmed by comparison with the experimental data [148]. In general, the exponent γ in thermodynamic scaling indicates the relative importance of the density and thermal energy in the slowing down of structural relaxation, being large for systems where density is important and low for systems where the energetic contribution dominates. Moreover, it has been demonstrated [141,149] that there is a connection between the *E_V_*/*E_P_* ratio and exponent *γ* (Figure 14). It is well-described by the following equation

*E_V_*/*E_P_* = 1/(1 + *α_P_Tγ*) (47)

where *α_P_* is the isobaric thermal expansion coefficient, and the solid line corresponds to the dependence of (47) with the constant *α_P_T_g_ =* 0.18. The latter relation is in good agreement with the empirical Boyer–Spencer rule: *α_P_T_g_ =* 0.2 [150]. Thus, both parameters, *E_V_*/*E_P_* and *γ*, might reflect similar underlying physics.

### 3.4. Tests of Elasticity Model

**Figure 15:**
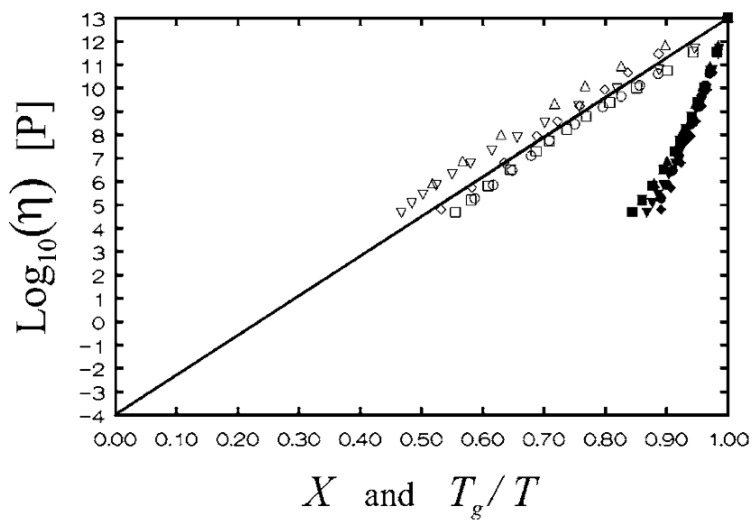
Viscosity as a function of inverse temperature (full symbols) and as a function of *X* = *G*_∞_(*T*)*T_g_/*G*_∞_(*T*_g_)*T (open symbols) for four organic liquids and one silicone oil. The approximate high-temperature limit of the viscosity is given at the lower left corner. Open symbols follow the diagonal line predicted by the shoving model, ending in the lower-left corner at a typical high-temperature viscosity. Reprinted figure with permission from [41]. Copyright (1996) by the American Physical Society.

In the shoving model of Dyre, the activation energy of the structural relaxation is connected to the infinite frequency shear modulus *G*_∞_(*T*) [13,43,44] (Equation (16)). An increase in *G*_∞_(*T*) upon cooling of a supercooled liquid leads to a super-Arrhenius behavior of its viscosity and structural relaxation time. However, for a quantitative comparison of experimental data to the model predictions, one needs to understand the exact meaning of *G*_∞_(*T*). In particular, should it be measured at a true infinite frequency (microscopic vibrations) or just at frequencies above the main structural relaxation? Recent molecular dynamic simulations [151] and experimental analyses [152] showed that the correct value of the instantaneous shear modulus *G*_∞_(*T*) of the shoving model refers to the plateau modulus at frequencies lower than fast picosecond relaxation but higher than structural relaxation. An analysis of several glass-forming liquids indeed revealed good agreement with the predicted behavior (Figure 15).

**Figure 16:**
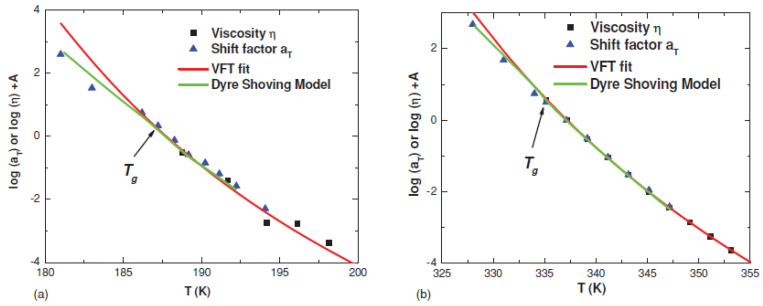
Temperature shift factors and dynamic viscosity for (**a**) m-toluidine and (**b**) sucrose benzoate. The dynamic viscosity data are vertically shifted by an arbitrary constant A to make the curves overlay. The green line represents the Dyre shoving model fit to the dynamic viscosity data and the stress relaxation shift factor *a_T_*. The red line represents the VFT fit to the dynamic viscosity and the stress relaxation *aT*. For m-toluidine, A = −8.7; for sucrose benzoate, A = −9.67. Reprinted from [153] with the permission of AIP Publishing.

A careful evaluation of the shoving model was recently done by McKenna and coworkers [153]. The glassy modulus *G*_∞_ was obtained by the extrapolation to zero time or infinite frequency of the Kohlrausch–Williams–Watts function to the experimental data for *m*-toluidine and sucrose benzoate. It was found that *G*_∞_(*T*) obtained from the KWW function provides a good description of the temperature-dependent dynamics of these liquids, even better than the classical VFT approximation (Figure 16). Thus, this model captures the non-Arrhenius behavior of structural relaxation in glass-forming liquids. Recently, an analysis of a large number (>100) of glass-forming liquids using the shoving model was compiled in Ref. [154]. For most materials, there is a good agreement with the experiment, although there are some materials that do not confirm the model. There is no systematic trend in chemistry in both groups of materials. Moreover, different authors may have different conclusions for the same material about the agreement of the elastic model with the experiment. Thus, at the moment, it is not clear what the limitations are of the applicability of the shoving model. Analyzing a large set of metallic glasses, it has been found that a better fit is achieved when the activation energy is a combination of shear and longitudinal *M*_∞_ moduli [152]:

1/*E* ∝ 2/*G*_∞_ + 1/*M*_∞_ (48)

This relationship has been justified by considering the contribution of shear and longitudinal modes to the total mean-squared atomic displacements [152].

Another interesting relationship between the fragility of glass-forming liquids and their mechanical properties at *T_g_* was suggested in [14,155]. It has been shown empirically that the ratio of the high-temperature activation energy of the viscous flow to *T_g_* correlate with the inverse fragility:

*m* ∝ *T*_*g*_/*E*_∞_ (49)

It is known that *E_∞_* is determined by the infinite frequency shear modulus *E_∞_* *∝ G_∞_* [43], see, e.g., the shoving model. On the other hand, *T_g_* is also proportional to the elastic constants ([14] and references therein). Thus, from Equation (49), one should expect that

**Figure 17:**
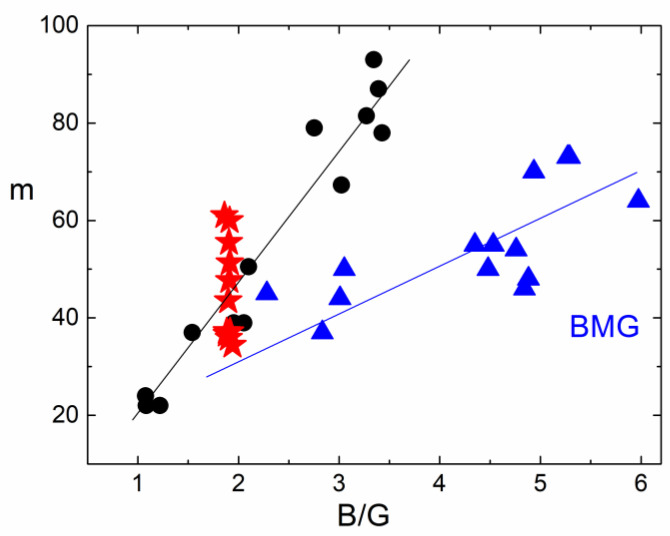
Comparison of m vs. *B*/*G* dependence in chemically simple nonmetallic glasses (circles) and bulk metallic glasses (triangles). Stars show the B_2_O_3_–Li_2_O mixture with different compositions and provides a clear illustration of the deviation in chemically complex systems. Data from [14,156].

*m* = *const* + *αB*/*G* (50)

where *B* is the bulk modulus. Since *m* is defined as *T_g_*, we can approximately consider infinite frequencies *B* and *G* as elastic moduli in the glassy state or just at *T_g_*. This correlation between *m* and *B*/*G* was indeed confirmed for many chemically simple nonpolymeric glass formers [14,155] with best fit parameters of *α* = 29 and *const* = −12 (Figure 17). Interestingly, the minimum possible fragility *m*_0_ = 17 would correspond to *B* ≅ *G*.

When the glass-forming liquid is of a more complex nature, the correlation (50) might not work, or the parameters may be different. We know three types of systems with such deviations: (i) bulk metallic glasses (BMG) [156], (ii) polymers with a strong dependence of fragility on the molecular weight [156], and (iii) many-component (chemically complex) systems [156,157] such as alkaline silicate or borate glasses. Polymers will be considered in a special section below, and let us briefly consider the possible reasons for the deviations in two other classes of glass-forming systems.

The specific feature of BMG that distinguishes them from nonmetallic glasses is a free electron gas. It gives a large contribution to the bulk modulus but, as any gas, does not contribute to the shear modulus. As a result, the lattice contribution to the bulk modulus is only a part of the total measured B. In this case, the coefficient α in Equation (50) should be smaller than in nonmetallic glasses. This agrees with the experimental data (Figure 17) [156].

It was shown [14] that, in multicomponent systems such as, e.g., lithium borate or silicate glasses, fragility is higher than one can expect from Correlation (50) (Figure 17). Later, in Ref. [157], the authors collected more data on multicomponent systems that confirm the same property—in all these systems, fragility is always higher than expected according to Correlation (50). In other words, all points corresponding to multicomponent systems would lie above the correlation line for chemically simple glasses (Figure 17). Apparently, increased fragility is a characteristic feature of many-component liquids and might be affected by the entropy of the mixture. A good example is decalin: the pure cis-decalin has a fragility *m*~60–70 [99,158], while the usual decalin is a mixture of approximately 50–50 cis- and trans-components and has an anomalously high fragility of *m*~146 [159,160].

Concluding Section 3, we want to emphasize that all three approaches, thermodynamic, free volume, and elastic models, describe temperature variations in structural relaxation reasonably well, and all have some problems. The analysis of various experimental data clearly indicates the absence of the divergence of the characteristic length scale of cooperativity/heterogeneity expected in entropic models. This analysis also demonstrates that density is not the only parameter that controls structural relaxation, and a purely thermal contribution can dominate in some liquids, e.g., glycerol. Elastic models look attractive and easy to understand but require more microscopic justification.

## 4. Connection between Fast and Slow Dynamics

Many experimental studies revealed strong correlations of fast dynamics, even in the glassy state, with fragility of the liquid state [14,43,48,49,161,162,163]. This apparent connection between dynamics on the ps time scale and the temperature dependence of the structural relaxation on the time scales of seconds and minutes remains a great puzzle [164] and is the focus of this section.

### 4.1. Relationship of Fragility and Short Time 〈u^2^〉

Already, in 1992, Buchenau and Zorn discovered [48] that the temperature dependence of MSD on a time scale faster than ~1 ps in selenium correlates well with the behavior of its viscosity: log*η*(*T*) ∝ 1/〈*u*^2^(*T*)〉 for over more than 15 decades in variations in *η*(*T*). This relationship is predicted by the elastic model of the glass transition (Equation (18)) [43]. As we discussed above, ETGF [45,70,71] and Wyart’s [78,79] models also suggest a connection between entropy and MSD. In addition, the experimental studies revealed that the free volume measured by the positron annihilation (PALS) technique and 〈*u*^2^(*T*)〉 have a similar *T*-dependence [48,165,166]. This observation suggests that MSD on the ps time scale can provide a qualitative measure of the free volume and provide another justification for the connection between log*τ_α_*(*T*) and 1/〈*u*^2^(*T*)〉, as recently discussed in Ref. [167].

**Figure 18:**
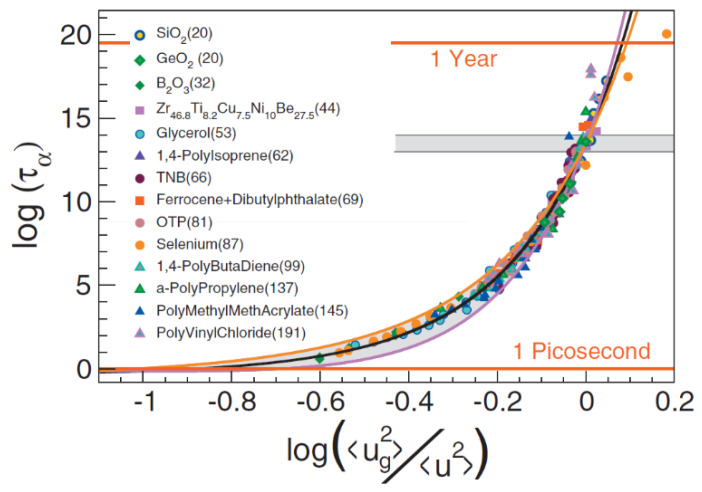
Scaling of the structural relaxation time *τ_α_* (in MD units) vs. the reduced mean square amplitude 〈*u*^2^(*T_g_*)〉/〈*u*^2^(*T*)〉. The grey area marks the glass transition. The continuous black line is Equation (51) shifted vertically by 10.498 to compile with the MD units. The numbers in parentheses denote the fragility *m*. Reprinted from [169] with the permission of AIP Publishing.

An improvement of Expression (18) was suggested in Refs. [168,169]. The authors took into account the local spatial heterogeneity in MSD [168,169]. Describing the fluctuations of the parameter *λa*^2^ in Equation (18) by the Gaussian distribution function and averaging over the volume, an improved expression was obtained:

log *τ*_α_ = *a*_0_ + *a*_1_〈*u*^2^(*T_g_*)〉/〈*u*^2^(*T*)〉 + *a*_2_(〈*u*^2^(*T_g_*)〉/〈*u*^2^(*T*)〉)^2^ (51)

where *a*_0_ = −10.922 (assuming *τ_α_*(*T_g_*) = 10^3^ s), *a*_1_ = 1.622, and *a*_2_ = 12.3 are universal constants. The authors analyzed the relationship between 〈*u*^2^〉 on the ps–ns time scale and $\;\tau_{\alpha }\left(T\right)$ using a wide range of materials with fragility varying from *m*~20 to *m*~190. The proposed universal expression (Equation (51)) indeed makes a good scaling plot for all glass formers analyzed in [168,169] (Figure 18). The MD simulations showed [151] that even the dynamics of completely flexible unentangled polymer melt follow this universal scaling.

Another generalization of the relation between log *τ*_α_ and 〈*u*^2^(*T*)〉 was suggested in Ref. [170]:

(52) $$\log(\tau_{\alpha }\left(T\right)/\tau_{0})={\left(\frac{u_{0}^{2}}{\langle u^{2}\left(T\right)\rangle }\right)}^{\alpha /2}$$

The exponent *α* is a measure of the free-volume anisotropy. For the spherically symmetric case, α = 3, this function also gives a good master plot for various materials with α in the interval of 3 ÷ 5.5 [170]. The ECNLE theory of Schweizer and coworkers also predicts a conceptually similar relationship, log*τ_α_/τ*_0_ ∝ 1/(*r_loc_*)^2^ [88], where *r_loc_* is the transient localization length, which quantifies the transient localized-state Debye–Waller factor.

These interesting empirical observations suggest that the degree of non-Arrhenius temperature dependence of *τ_α_*(*T*) (fragility) is directly related to the anharmonicity in the temperature behavior of 〈*u*^2^(*T*)〉*:* a strongly anharmonic 〈*u*^2^(*T*)〉 corresponds to a very fragile system and almost harmonic 〈*u*^2^(*T*)〉 to strong systems.

Indeed, it was shown [171] that fragility correlates with anharmonicity. Moreover, in Ref. [52], molecular dynamic simulations of the glass transition in binary Lennard–Jones systems revealed that the kinetic fragility of the system, as well as the nonexponentiality of the relaxation, depends on the anharmonicity of the interatomic potential. In more accurate terms, this work shows that fragility depends on the “openness” of the potential, i.e., on how slow the attractive part of the interparticle potential increases with the distance.

### 4.2. Fragility and Fast Dynamics

**Figure 19:**
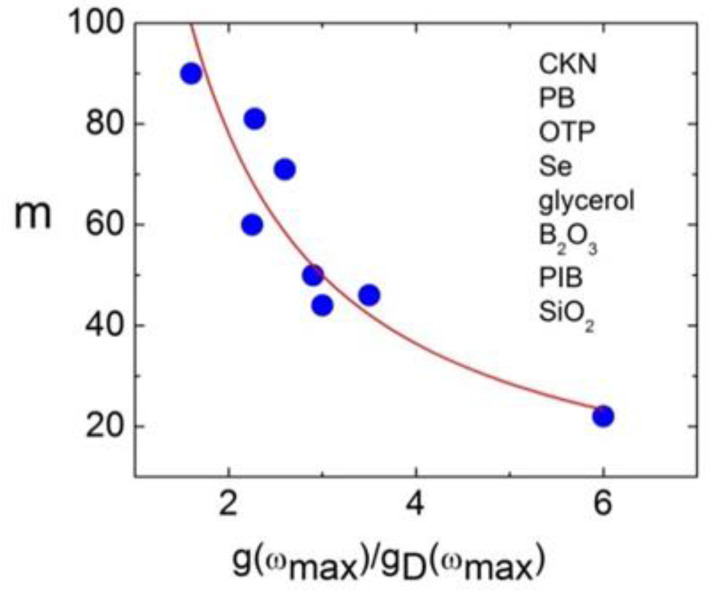
Correlation of fragility with the boson peak amplitude measured in units of the Debye density of the states *g_D_*(*ω*). The line is *m*∝*g_D_*(*ω_max_*)/*g*(*ω_max_*), and *ω_max_* is the frequency of the boson peak maximum. Data from Ref. [14].

MSD on the ps time scale in supercooled liquids and glasses is defined by the fast picosecond dynamics that have two distinct features: the vibrational boson peak at about 1 THz and the fast *β*-relaxation at lower frequencies that overlaps with the low-frequency part of the boson peak. It has been shown that the ratio of the boson peak amplitude to the expected Debye vibrational density of the states can be as large as 5 ÷ 7 in one of the strongest glasses: silica and decreases strongly in more fragile glasses [14]. An analysis of a number of glass formers indeed revealed that the amplitude of the boson peak estimated from the neutron scattering data in the glassy state correlates with the fragility (Figure 19) [14].

**Figure 20:**
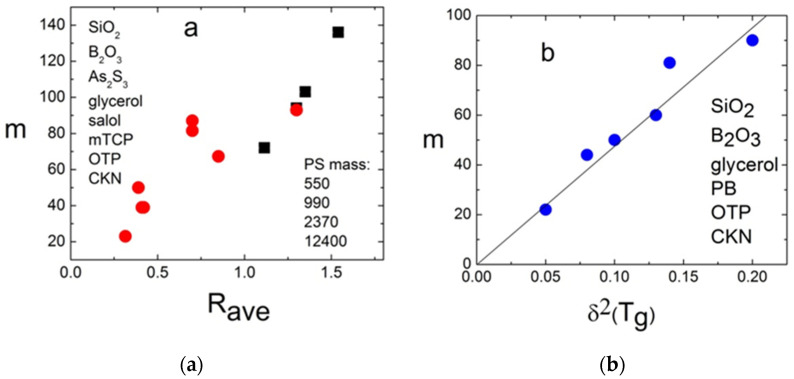
(**a**) Correlation of fragility with the parameter *R* for some inorganic glasses (circles, listed in increasing fragility order) and for polystyrene with different molar mass (squares, molecular weight = 550, 990, 2370, and 12,400 in the order of increasing fragility). (**b**) Correlation of fragility with the parameter *δ*^2^(*T_g_*) that characterizes the ratio of integrated intensity of the fast relaxation to the integrated intensity of the boson peak. Data from Ref. [14].

The intensity of the fast relaxation in various models was connected to the density of the asymmetric double–well potentials [172,173], concentration of the free volume [174,175], the nonergodicity parameter [163], and to the anharmonicity of the lattice [176,177]. It appears that the fast relaxation also correlates with fragility but in the opposite way: the higher the fragility, the stronger the fast relaxation contribution relative to the vibrational contribution at *T_g_*. Two parameters were used to quantify this correlation. In Ref. [161], it was suggested to use parameter *R*, which is the ratio of the intensity of scattering light or neutrons in the minimum between the fast relaxation and the boson peak at *T_g_* to the maximum of the boson peak at the same *T*. This is an easy and model-independent method to measure the intensity of the fast relaxation relative to the amplitude of the boson peak. We note that, for some very fragile glass formers, the fast relaxation is so strong that, at *T_g_*, there is no minimum between the fast relaxation and the boson peak, which makes such an analysis less reliable. A more accurate but more complicated parameter *δ*^2^ is the ratio of the integral over the fast relaxation spectral density to the integral over the boson peak [176]. A correlation of both parameters with fragility is shown in Figure 20.

So far, there is no clear model connecting fragility and amplitude of the fast relaxation. A qualitative explanation can be formulated based on the contributions of vibrations and fast relaxation to the total MSD [161]: 〈*u*^2^(*T*)〉 = 〈*u*^2^(*T*)〉_vib_ + 〈*u*^2^(*T*)〉_rel_. The first term has essentially harmonic behavior 〈*u*^2^(*T*)〉_vib_ ∝ *T*, while the second varies much stronger with *T*. In that case, using the relationship between log*τ_α_* and 〈*u*^2^〉 (Equations (18) and (51)) one can expect almost Arrhenius-like behavior for *τ_α_*(*T*) in liquids where MSD is dominated by the vibrational contribution and strongly super-Arrhenius for systems where the fast relaxation contribution dominates MSD. This explains why systems with the boson peak dominating the spectra of the fast dynamics exhibit strong behavior, while liquids with strong and fast relaxation have high fragility (Figure 20).

### 4.3. Fragility and Nonergodicity Parameter in Glassy State

An interesting correlation between the high-frequency property of the glassy state and fragility of the liquid was found empirically in Ref. [163]. The authors analyzed the nonergodicity parameter *f*(*Q*,*T*) in the glassy state obtained from the intensity of the Brillouin lines, measured by inelastic X-ray scattering. In the low *Q* limit, the temperature dependence of *f*(*Q*,*T*) in the glassy state can be approximated by a simple relationship [163]:

(53) $$f\left(Q\to 0,T\right)=\frac{1}{1+\alpha \frac{T}{T_{g}}}$$

**Figure 21:**
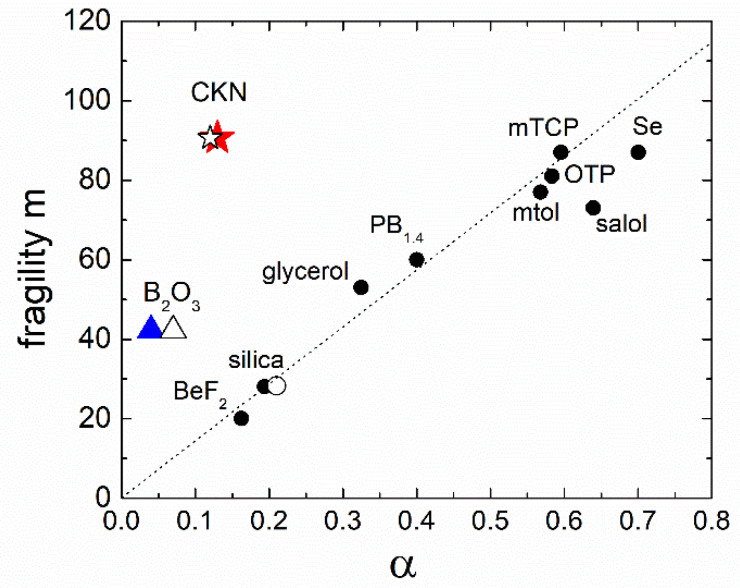
Correlation between the parameter *α* taken from Ref. [163] and fragility *m* (solid circles). X-ray and light scattering data for B_2_O_3_ (solid and open triangles, respectively) and CKN (solid and open stars, respectively) and light scattering data for SiO_2_ (open circle) are added. This figure is from Ref. [14].

An analysis of several glass-forming systems revealed [163] a good correlation between fragility of a liquid and the parameter *α* that characterizes the temperature variation of *f*(*Q*,*T*) in the glassy state (Figure 21). Although some systems deviate from this correlation (CKN and B_2_O_3_ in Figure 21) [14,178], it is puzzling how the temperature dependence of the THz vibrational dynamics in glass can predict the temperature dependence of structural relaxation in a liquid. Essentially, it suggests that the nonergodicity parameter (the amplitude of the structural relaxation) at *T_g_*, *f*_0_(*T_g_*)~1/(1 + *α*), correlates with the liquid fragility. In Ref. [14], the authors noted that the correlation of *m* and *α* is connected to the above-discussed correlation between *m* and the Poisson’s ratio of the respective glass. The parameter α in some approximation can be expressed in terms of the ratio of the bulk and shear moduli of the glass or, equivalently, in terms of Poisson’s ratio.

In addition to CKN and B_2_O_3_, several polymers deviate strongly from the proposed correlation of *α* and *m* [179]. The authors of [179] argue that the deviation is related to the difference between *T_K_* and *T*_0_ and can be ascribed to an additional contribution to the configurational entropy (e.g., secondary relaxation), which does not affect the structural relaxation and fragility. By correcting the nonergodicity parameter, they returned the deviating systems back to the proposed correlations. This explanation, however, has some shortcomings, which we will discuss later in Section 5.2 of this review.

### 4.4. A General Picture Connecting Fast and Slow Dynamics

Based on the above discussion, we can try to formulate a general picture relating fast dynamics to the temperature dependence of the structural relaxation. It is obvious that the structure and dynamics are determined by the interatomic/intermolecular interactions. In liquids with directional bonds (e.g., covalent- and H- bonding systems), there is a significant shear modulus *G*_∞_ (relative to the bulk modulus) even at high temperatures. As a result, the shear modulus does not increase much with *T* upon cooling, and (following the shoving model) the structural relaxation in these liquids show only a slightly super-Arrhenius behavior. In contrast, in liquids without directional interactions (e.g., VdW and ionic systems), the shear rigidity appears mostly due to jamming and is very low (relative to the bulk modulus) at high *T*. This leads, however, to a strong increase in the shear modulus upon cooling due to the jamming of particles. As a result, the activation energy of the structural relaxation increases strongly upon cooling, and these liquids exhibit strong super-Arrhenius temperature behavior (high fragility). This picture is also consistent with the observed temperature variations in *S*(*Q*) [134]: these variations are very weak in strong systems, while they are significant in fragile glass-forming systems (Figure 9).

It is not obvious how to connect this elasticity picture to change in the configurational entropy. In a simple naïve picture, it seems obvious that more rigid systems (higher modulus) should have less accessible conformational states. Thus, the configurational entropy should vary much faster with the temperature in systems that exhibit strong temperature variations in *G*_∞_(*T*) than in systems where *G*_∞_ is barely changing with *T*, and there should be a connection between the entropic and elastic models. However, this connection is still not well-explored and understood.

The same interatomic potential obviously determines the fast dynamics. Systems with direct bonds have better-defined positions of atoms and less frustration in their packing. This leads to a smaller amplitude of fast picosecond relaxation (rattling in the cage), i.e., to a smaller amplitude of 1-*f*_0_ and, accordingly, to a larger nonergodicity parameter *f*_0_. The latter determines the amplitude of the structural relaxation and, in particular, the amplitude of the elastic constant fluctuations that can be considered frozen on the time scale of the fast dynamics. This leads to an increase in the boson peak amplitude (relative to the expected Debye model) in materials with higher *f*_0_. In contrast, systems without direct bonds have much larger frustration in their packing and much higher amplitude of the rattling in the cage. This leads to a much lower *f*_0_, a weaker boson peak, and a higher amplitude of the fast relaxation.

This consideration explains the connection between the relative contributions of the fast relaxation and the boson peak to fragility (Figure 16 and Figure 17). It also relates both properties to the nonergodicity parameter f_0_. Moreover, it also explains well the correlation between the behavior of MSD and structural relaxation time with the temperature. According to the above picture, *f*_0_(*T*) is large in liquids with direct bonds and does not change much with the temperature. As a result, *G*_∞_(*T*) also shows rather weak temperature variations. This leads to an almost Arrhenius behavior of *τ_α_*(*T*), a strong boson peak, and a low amplitude of the fast relaxation. The data for B_2_O_3_ indeed show that the boson peak dominates the fast dynamics spectra of this relatively strong liquid even at temperatures of ~2.5*T_g_* [180]. On the contrary, systems with no directional bonds have a lower value for *f*_0_(*T*), which varies strongly with *T*. This leads to a strong variation in *G*_∞_(*T*), to a strongly non-Arrhenius behavior of *τ_α_*(*T*), a weak boson peak, and a high amplitude of the fast relaxation. Data for the fragile system CKN show that fast relaxation dominates its fast dynamics spectra even at *T_g_*, in full agreement with this scenario [161]. This qualitative consideration provides some simple picture connecting many pieces of the puzzle, including the role of elasticity, shear modulus, and interatomic interactions (directional/nondirectional) in fragility, the nonergodicity parameter, and fast dynamics of glass-forming systems.

## 5. Extreme Fragility in Polymers

The chain connectivity in polymers leads to an additional relaxation process, chain relaxation. It appears on the time scale longer than the structural relaxation. The latter in polymers is called segmental relaxation [181]. Chain relaxation controls the viscosity, while glass transition is controlled by segmental relaxation. There is numerous experimental evidence that chain relaxation in polymers has a different temperature dependence than segmental dynamics [182]. Thus, it is incorrect to use polymer viscosity data to discuss the steepness of the temperature dependence of the structural (segmental) dynamics, and we will focus the next section on the discussion of segmental dynamics only.

### 5.1. Failure of Many Correlations in Polymers

**Figure 22:**
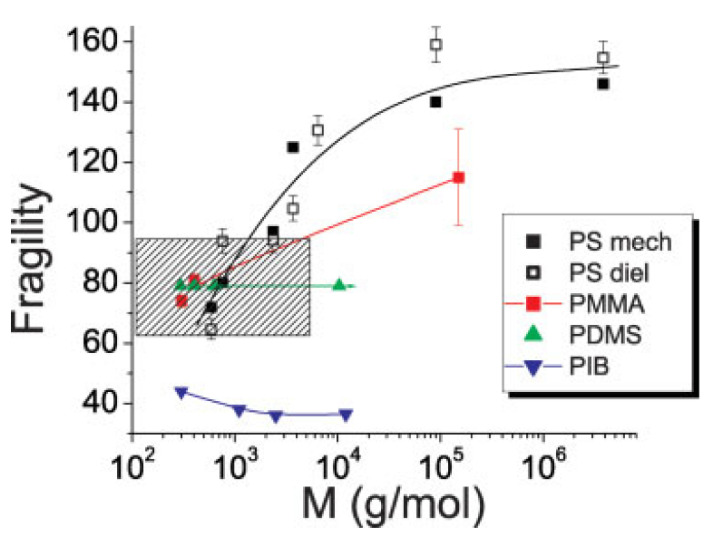
Molecular weight dependence of fragility in various polymers. The shaded area represents the region characteristic of small molecules. The fragility values for PIB are those calculated from dielectric spectroscopy. Reprinted figure with permission from [187]. Copyright (2008) by John Wiley & Sons, Inc.

The fragility of small molecular systems usually does not exceed 100, while there are many polymers with *m* > 150 and even reaching *m*~200 [5,6]. Therefore, polymers exhibit extremely high fragility, significantly above the value of m typical for small molecules, and this fact deserves special discussion. The mechanism of extremely high fragility of polymers is apparently related to chain connectivity. In contrast to small molecules, polymers have an additional parameter that affects their structural dynamics—the chain length, i.e., molecular weight (MW). The glass transition temperature increases strongly with the increase in MW for most of polymers with nonpolar chain ends. Detailed studies revealed that the fragility also increases with MW in most polymers [183,184,185,186]. Very flexible polymers, such as poly(dimethyl siloxane) (PDMS) and polyisoprene (PIP), show a very weak increase of fragility with MW [183]. They also have a relatively weak change of *T_g_* with MW [183]. However, polymers with a more rigid structure, such as polystyrene (PS) and poly(methyl methacrylate) (PMMA), show a much stronger increase in the fragility with MW (Figure 22). This increase in the fragility with MW and its dependence on chain rigidity is captured by both ETGF and Schweizer’s theory. We are aware of only two polymers where the fragility decreases with an increase in MW, polyisobutylene (PIB) [187], and poly(ethylene acrylate) (PEA) [188]. We want to stress that PIB actually shows many other unusual-for-polymers properties [187].

**Figure 23:**
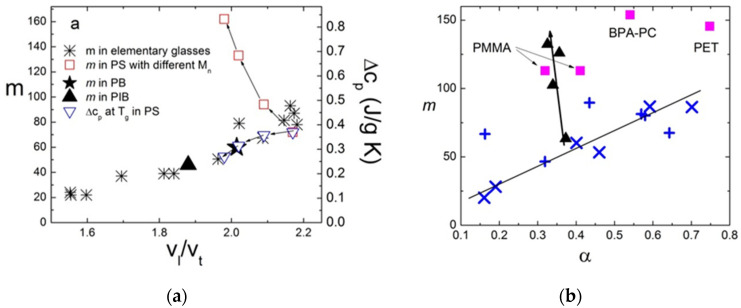
(**a**) Fragility vs. *v_l_*/*v_t_* for simple liquids (stars) and some polymers. Polybutadiene (PB, star) and polyisobutylene (PIB, solid triangle) follow the correlation of simple liquids independently of the molecular mass, while polystyrene (PS, open squares) agrees with the correlation for small molecular weights (MW = 550 and 990 g/mol) and strongly deviates with increasing molecular weights (MW = 8000 and 220,000 g/mol). Interestingly, the jump of the specific heat at *T_g_* in PS with different MW is in a good agreement with the correlation (open triangles, right axis). Data from Ref. [14]. (**b**) Correlation between the nonergodicity parameter α and fragility for different molecular liquids and for PS samples at different molecular weights (solid triangles) [190]. The arrows indicate the direction from shorter chains to longer chains. Crosses represent *α* calculated from IXS data in other studies. Squares—data for polymethyl methacrylate (PMMA), bisphenol A polycarbonate (BPA-PC), and polyethylene terephthalate (PET) from Ref. [179].

It has been demonstrated that correlations of fragility with various other material properties established for small molecules fail in the case of many (but not all) polymers. This includes the following correlations: (i) ln*τ* vs. 1/*S_c_T* [189], (ii) Δ*C_p_* vs. *m* (Figure 23), (iii) *K*/*G* (or *v_l_*/*v_t_*) vs. *m* (Figure 23), and (iv) non-ergodicity parameter vs. *m* (Figure 23). For example, it has been shown in [189] that lnτ follows variations of configurational entropy in flexible polymers, such as PDMS and PIP, while strong deviations appear in rigid polymers, such as PS and PMMA. The same seems to be true for the other listed correlations. It is interesting to note that, in most of these cases, the correlations hold for oligomers (very short chains) but fail progressively with the increasing molecular weight (Figure 23). The data for monomers and oligomers of the same polymers agree well with the correlations established for small molecules (Figure 23). However, the increase in MW in these polymers leads to a significant increase in m, while many other properties (e.g., *K*/*G* and nonergodicity) remain essentially not affected by the MW.

This MW dependence questions the idea proposed in [179] that ascribes the failure of the correlations in polymers to their secondary relaxations. First of all, secondary relaxations are also known for small molecules, where the correlations hold. Second, the secondary relaxations exist in short and long polymers and show rather weak MW dependence. Thus, according to this idea, the failure of the correlations should not depend strongly on MW. This expectation clearly contradicts the experimental data (Figure 23). In contrast, the failure of the correlations might be explained by a strong increase in fragility due to some intramolecular polymer-specific mechanism that does not influence other properties. Indeed, polymers with a weak MW dependence of fragility (e.g., PIP, polybutadiene (PB), and PDMS) follow well the correlations established for small molecules. Only polymers with a strong increase in fragility with MW (e.g., PS and PMMA) deviate from these correlations, and we will discuss this point in more detail below.

### 5.2. Polymer Specific Contribution to Fragility

**Figure 24:**
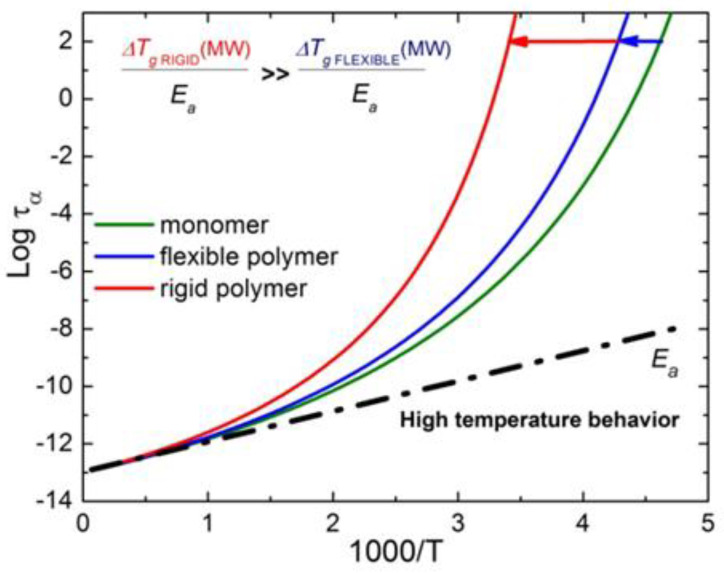
Schematic presentation of the influence of molecular weight on the temperature dependence of the structural relaxation in polymers. At high temperatures, the behavior is rather MW-independent. An increase in the molecular weight results in a stronger slowdown of the structural dynamics as the temperature is lowered, i.e., the increase of *T_g_*. As a result, the temperature variations of *τ_α_* appear to be steeper, i.e., more fragile. This slowdown is weaker in flexible polymers and more significant in rigid ones.

**Figure 25:**
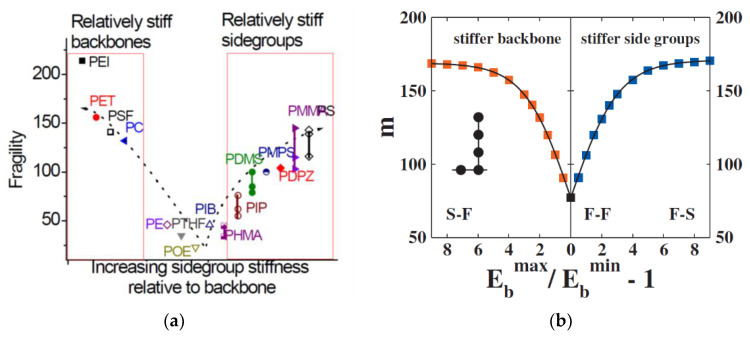
(**a**) Schematic presentation of polymers classification based on the relative flexibility of the side groups and backbone. It is observed that polymers tend to be more fragile as the flexibility of the side groups becomes different from that of the backbone (Figure adopted from Ref. [198]). (**b**) Theoretical prediction for the fragility of polymer melts as a function of the relative backbone and side group rigidity, expressed as the bending energy ratio. Reprinted from [199] with the permission of AIP Publishing.

To understand the microscopic mechanisms specific for polymers with high fragility, let us discuss the qualitative picture proposed in [190] (Figure 24). Let us assume that the temperature dependence of *τ_α_*(*T*) can be well-described by a single VFT. Then, the high-temperature behavior is expected to be rather independent of MW, because the chains are very flexible at a high *T*, and the chain length has little effect on the local segmental dynamics. In that case, the VFT parameters *τ*_0_ and *B* should have rather weak MW dependence, and the main change is caused by the effective MW dependence of *T*_0_(MW). In the case of flexible chains, the shift of *T*_0_ and *T_g_* with MW is very small, while, in rigid chains, it will be very strong. As a result, flexible chains have a weak dependence of *T_g_* and fragility on the MW, while rigid chains exhibit much a stronger dependence of both *T_g_* and fragility on the molecular weight (Figure 25). This qualitative picture also suggests that segmental dynamics in polymers with different molecular weights should scale universally with *T-T_g_*(MW). This scaling indeed has been reported for a number of polymers [191,192,193,194].

The generalized entropy theory of glass formation (ETGF) discussed above [45,70,71] provides predictions for the molecular weight dependence of *T_g_* and fragility that agrees qualitatively with the experimental results. It also predicts that the fragility should increase with *T_g_, m* ∝ *T_g_*, if the cohesive energy remains the same. This result is indeed observed for many, although not all, polymers [183,195,196]. Qualitatively, ETGF relates the polymer fragility to frustration in their packing [45]: the higher the frustration in packing, the higher the fragility. This qualitative picture agrees well with the experimental data and explains why flexible polymers show a weak or no dependence of fragility on the molecular weight, while rigid polymers exhibit a strong dependence of fragility on the MW (Figure 22). A detailed analysis of the experimental data for many polymers indeed confirms that the chain rigidity and bulkiness of the side groups play important roles in polymer fragility [184] (Figure 25), in good agreement with ETGF (Figure 25). Moreover, it has been found that the density of PIB at *T_g_* actually increases with the MW [187], a trend unusual for polymers. Thus, the packing in PIB improves with the MW, and this might explain the decrease in fragility with the MW observed in this polymer [187] (Figure 22), again in agreement with the prediction of ETGF.

Detailed studies also demonstrated that intermolecular interactions affect significantly both *T_g_* and the fragility of polymers [197]. It has been found that the polar (interacting) groups attached directly to the backbone increase both the *T_g_* and *m*, but if they are attached to a side group, the direction of changes in the fragility is not obvious. The question that has not been really studied in detail is the role of chain tacticity in polymer fragility. Many polymers have different isomers due to the positions of their side groups that might contribute additionally to the configurational entropy of the system. It is known that polymer tacticity leads to a change in *T_g_*. Whether it also plays any significant role in fragility remains an open question.

**Figure 26:**
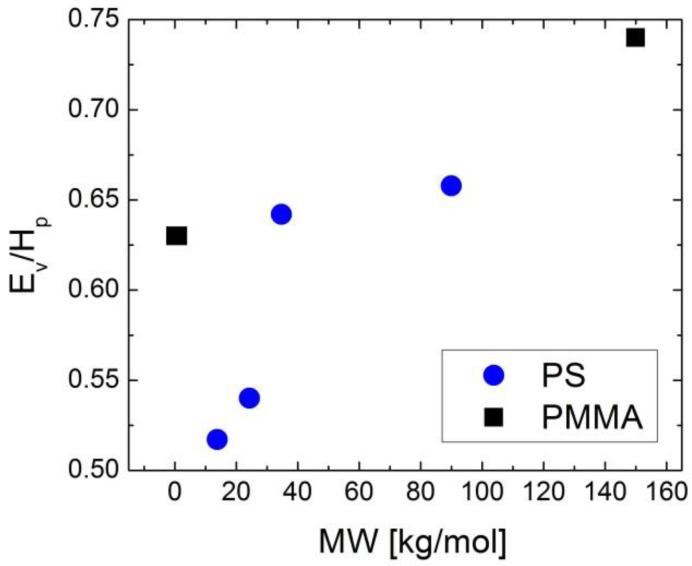
Variations of the activation enthalpy ratio with the molecular weight for polystyrene (circles) and polymethylmethacrylate (squares). The data for PS are from Ref. [185] and, for PMMA, are recalculated from Ref. [186].

For many decades, it was accepted that free volume is the major mechanism controlling polymer dynamics. This is based on the strong dependence of polymer dynamics on pressure. However, a detailed analysis demonstrated that the energetic contribution actually dominates the temperature variations of segmental dynamics in many polymers, including extremely fragile ones [135]. It is obvious from the value *E_V_*/*E_P_* > 0.5 and relatively small value of the thermodynamic scaling exponent γ for most of the polymers [135] (see also Section 3.3). Moreover, the study in PS revealed a strong increase in the ratio *E_V_*/*E_P_* with the molecular weight (Figure 26). A decrease of the exponent *γ* with an increase in the MW was also reported for PMMA [186]. Thus, the roles of the free volume and density actually decrease with the increasing of their molecular weight.

All these results suggest that intramolecular energy and entropy contribute significantly to the behavior of the polymer segmental relaxation, and this might lead to a polymer-specific contribution to fragility. This point has been recently emphasized by Colmenero [200]. An analysis of the MD simulation results clearly suggests that an increase in the chain rigidity, energy barrier for torsion rotation, and chain bending leads to a strong increase in *T_g_* and fragility [200,201]. These intramolecular polymer-specific contributions to fragility are probably at the core of the failure of the correlations discussed in Section 5.1. These correlations work well for small molecules and oligomers and apparently reflect the role of intermolecular interactions in fragility and other properties, e.g., the modulus, entropy, nonergodicity parameters, etc. Apparently, intramolecular degrees of freedom in rigid polymers lead to a strong rise in fragility, while they do not significantly affect their other properties. This analysis emphasizes that glass transitions in relatively rigid polymers have specific behaviors not observed in low molecular weight liquids.

**Figure 27:**
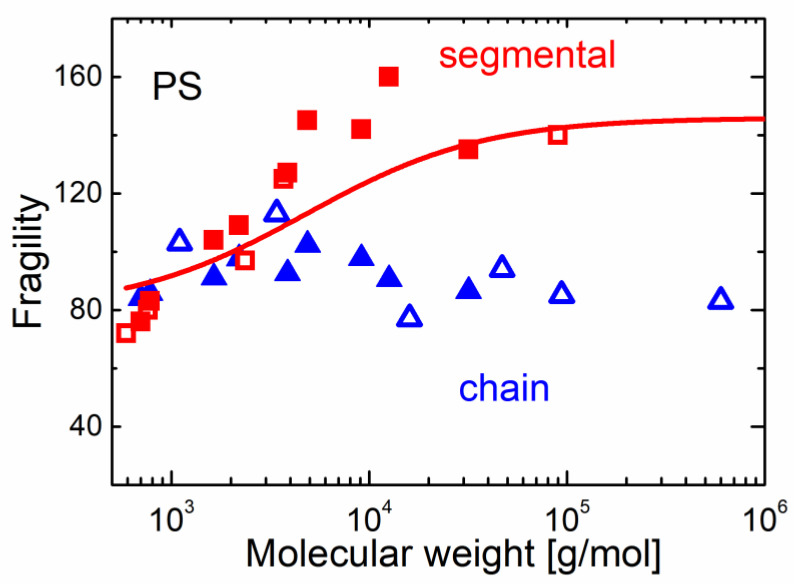
Molecular weight dependence of fragility for segmental relaxation (red squares) and viscosity (blue triangles) in PS. Solid symbols—data from Ref. [202] and open symbols—data from Ref. [204]. Fitting line according to the model from Ref. [202]. Adapted with permission from [202]. Copyright (2018) American Chemical Society.

Another idea concerning this problem was presented in Ref. [202]. The analysis in this paper revealed some scaling of segmental relaxation and viscosity as a function of the temperature in polymers with various molecular weights, MW. Viscosity falls on a master curve when plotted vs. *T_g_*(MW)/*T*. Since the viscosity is determined by chain relaxation, this master curve means that chain fragility is basically the same for polymers of different molecular masses. The segmental relaxation time falls, on the other hand, on the master curve as a function of *T-T_g_*(MW), which is consistent with earlier reports [191,193,194,203]. It is easy to show formally that the slope of the log*τ_α_* vs. *T_g_*/*T* curve at *T_g_*, i.e., fragility, differs from the slope of the curve log*τ_α_* vs. *T-T_g_* by the factor *T_g_*. Thus, the existence of a master curve log*τ_α_* vs. *T-T_g_* means that the steepness of the temperature dependence of *τ_α_* vs. *T_g_*(MW)/*T* (fragility) should increase with the molecular weight approximately at *T_g_*(MW). This indeed happens in rigid polymers such as PS or PMMA (Figure 27).

The authors of [202] speculated that the observed difference in temperature scaling for chain and segmental relaxation can be explained by the fact that chains are not yet fully relaxed on the segmental relaxation time scale. This corresponds to the nonergodic behavior of polymers on this time scale, while the ergodicity is restored on the global molecular (chain) relaxation time scale. As a result, the configurational entropy that controls the segmental relaxation decreases with the cooling faster compared to the total configurational entropy that controls molecular-scale relaxation. Moreover, the fraction of configurational entropy that is not accessible on the time scale of segmental relaxation increases with the chain length, resulting in an observed increase in the segmental fragility with the MW. This explains the very high segmental fragility in many polymers, which is not observed in small molecule systems.

## 6. Quantum Effects and ‘Super-Strong’ Behavior of Water

Quantum effects, such as zero-point vibrations and tunneling, are usually negligible at glass transition. However, several recent papers analyzed a possible role of quantum effects in glass transition [205,206,207,208]. It was shown that, in materials with the ratio of *T_g_*/*T_D_* < 0.5 (*T_D_* is the Debye temperature), zero-point vibrations (quantum fluctuations) contribution to the mean-square atomic displacement (MSD) is larger than the thermal part [206]. This criterion can also be formulated in terms of the De Boer parameter, $\hbar /\sqrt{k_{B}a^{2}MT}$~0.1, where *M* is the molecular mass, and *a* is the interatomic distance. The estimates show that a significant influence of the quantum effects on glass transition may occur even at *T_g_*~50–100 K. It was argued [206,207] that the tunneling rate can be roughly incorporated into the usual expression for the thermally activated structural relaxation rate by including zero-point MSD in the total MSD in the elastic model of the glass transition. The authors of [206,207,208] used the generalized expression for the structural relaxation time Equation (51) with zero-point vibrations included into 〈*u*^2^(*T*)〉. According to this approach, a significant contribution of zero-point vibrations to the MSD should lead to very weak temperature variations in *τ_α_*(*T*) and, respectively, to an unusually low fragility of the system. It was also demonstrated [206,208] that the quantum effect may even lead to an apparent fragile-to-strong crossover in the temperature dependence of the structural relaxation time if the zero-point portion of the MSD is large enough.

In search for experimental evidence of the quantum effects in the glass transition, attention should be paid to materials with light molecules. The main problem is that light molecules (e.g., H_2_O, CH_4_, and NH_3_) crystallize easily. Thus, little is known about their glass transition. One of the promising materials is water with its light molecule and *T_g_* = 136 K. Various simulations of water have demonstrated that, even at ambient temperatures, quantum effects lead to an increase in diffusion coefficients and a decrease in relaxation times by ~15–50% [209,210]. Quantum effects should be even more pronounced at lower temperatures. An analysis of neutron scattering data in low-density amorphous (LDA) water shows that zero-point vibrations account for ~60% of the total MSD at *T_g_* [207,208]. Thus, one can expect a strong influence of quantum effects on the structural relaxation of water near its *T_g_*.

**Figure 28:**
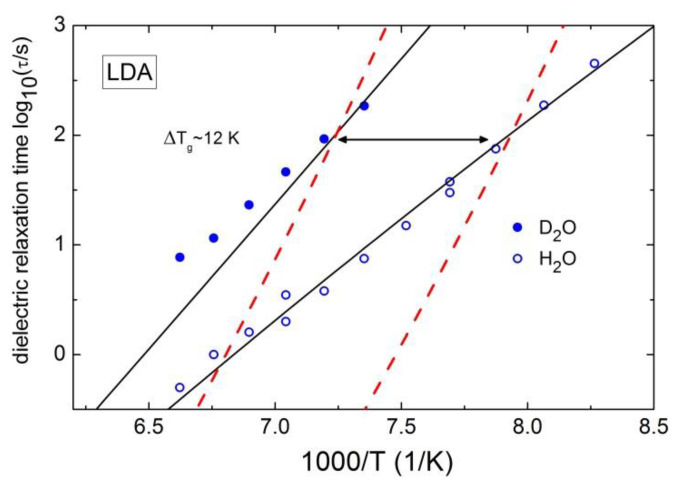
Temperature-dependent structural relaxation times *τ*(*T*) in LDA water. *τ*(*T*) increases about an order of magnitude when going from H_2_O (open symbols) to D_2_O (closed symbols). For liquids, this constitutes an unusually large isotope effect. The experimentally determined fragilities for LDA water are *m*_H2O_LDA_ ≈ *m*_D2O_LDA_ = 14 ± 1. The lines present the expected temperature dependence of *τ*(*T*) estimated from Equation (51) using the total MSD of LDA water (solid lines) and the MSD with zero-point vibrations excluded (dashed lines). The fragilities, estimated from the MSD data with zero-point vibrations taken into account, are: *m*_H2O_ ≈ 14.5 and *m*_D2O_ ≈ 19, similar to the experimentally determined values. When zero-point contributions to the MSD are excluded, the predicted fragility becomes *m*_H2O_ ≈ 37 and *m*_D2O_ ≈ 35. The calculations using LDA’s total MSD reproduce the temperature dependence of *τ*(*T*) well and thus emphasize the importance of quantum fluctuations in the dynamics of water at low temperatures. Data from Ref. [207].

Dielectric spectroscopy studies of LDA and vapor-deposited water [207,208] indeed revealed unusually slow temperature variations in the relaxation time. It leads to an unreasonable value of fragility *m* ~14, low activation energy *E_a_* ≈ 36 ± 1 kJ/mol (Figure 28), and unphysically long *τ*_0_ = 10^−11±0.3^ s. The obtained value of fragility is by far the lowest among the known liquids. Moreover, it is even lower than the expected minimum value, *m*_0_~17. This result is very surprising, because hydrogen bonding liquids usually have a fragility in the range of *m*~45–90. However, it agrees with what is expected for quantum effects behavior—abnormally slow *T*-dependence of the relaxation time.

**Figure 29:**
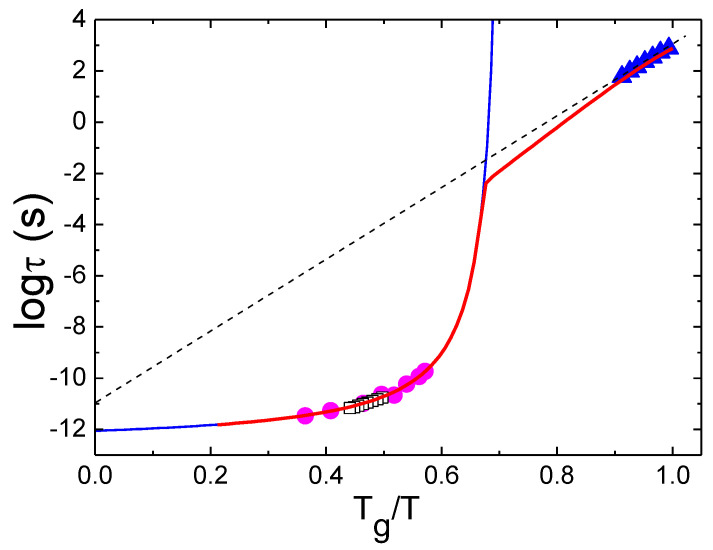
Comparison of low- (solid triangles) and high-temperature data for the structural relaxation time in water. Open squares—dielectric spectroscopy data in water [213] and solid circles—shifted viscosity data [214]. The dashed line presents an approximation of the low-temperature behavior by an Arrhenius dependence, and the solid blue line is an approximation of the high-temperature behavior using the Vogel–Fulcher–Tammann function. The solid red line presents a hypothetical transition line between low-temperature (quantum) and high-temperature (over barriers) regimes [208].

In order to verify the influence of the quantum effects on structural relaxation in water, *τ*(*T*) was estimated using Equation (51) with experimental MSD data, including zero-point vibrations. Indeed, the total MSD of LDA water reproduces experimental *τ_α_*(*T*) behavior surprisingly well (Figure 28). Thus, the anomalously low value of fragility obtained for the deposited water and for LDA might be the result of quantum effects in structural relaxation. This hypothesis was additionally confirmed by the anomalously large isotope shift of the water *T_g_* [207]. The substitution of hydrogen by deuterium in H-bonding liquids usually leads to a very small shift in *T_g_*, Δ*T_g_* < 1 K [207,211,212]. Recent dielectric studies, however, revealed a shift in *T_g_* between H_2_O and D_2_O Δ*T_g_*~10–12 K (Figure 28) [207]. As it was discussed in [207], this effect is consistent with the quantum effects and cannot be explained in the framework of classical relaxation. When increasing the temperature, the role of the quantum effects will fade. As a result, *τ_α_*(*T*) will be controlled by the usual thermally activated barrier crossing-type relaxation and return to a behavior typical for many liquids. This provides another explanation for the apparent fragile-to-strong crossover in the dynamics of water without involving any underlying liquid–liquid transition (Figure 29) [207,208].

Thus, taking into account the quantum effects in structural relaxation leads to two nontrivial consequences: (i) the fragility of the liquid can be much lower than the traditionally accepted limit of *m*_0_~17, and (ii) the temperature dependence of the structural relaxation can be sub-Arrhenius, i.e., the apparent activation energy can decrease upon cooling in contrast to the classical increase. The latter will lead to a rather low value of the *T_g_*/*T_m_* ratio [206]. We stress that the quantum effects are expected only in liquids of very light molecules, which also have *T_g_* significantly lower than the Debye temperature. Water is the lightest molecule that exists in the liquid state at ambient conditions and satisfies these criteria. It would be interesting to find whether quantum effects play any role in the glass transition and structural relaxation of other light molecules, such as methane and ammonia.

## 7. Conclusions and Perspectives

The microscopic mechanism of the strong slowing down of liquid dynamics upon approaching *T_g_* remains the focus of active research. A large body of experimental studies revealed that the steepness of the temperature dependence of structural relaxation (fragility) varies from *m*~14 in water to *m*~200 in some polymers. Usually, covalent-bonding liquids with strong directional bonds show a rather weak deviation of *τ_α_*(*T*) from an Arrhenius temperature dependence (low fragility), while most of the VdW and ionic liquids with nondirectional interactions exhibit strongly non-Arrhenius behavior (high fragility). Hydrogen-bonding systems usually have intermediate fragility, although there are some exceptions.

In this review, we specifically emphasized two important extreme cases of fragility. Polymers with extremely high fragility present a special case of glass-forming systems, which differs from small molecular liquids. This point was also emphasized in the review by Colmenero [200]. Chain connectivity, its intramolecular energy barriers and degrees of freedom, leads to a significant increase in polymer fragility, especially in rigid polymers. It is related mostly to an increase of the energetic part of the temperature dependence of structural relaxation (increase in *E_V_*/*E_P_*). A detailed microscopic picture of the polymer-specific contribution to fragility remains unresolved, although it is clearly stronger for more rigid chains, suggesting an important role of intramolecular energy barriers. The second extreme is presented by the unusually low fragility of water. It was ascribed to the quantum effect, and it would be important to verify this hypothesis in examples of other low-*T_g_* liquids of light molecules.

Entropic models have been rather successful in connecting the dynamics and thermodynamics, and there is indeed a good correlation between ln*τ_α_* and configurational entropy for many materials. However, the search for a diverging length scale and an increase in cooperativity of the structural relaxation (the core of the entropic models) so far did not find any clear confirmation. The experimental studies suggest rather weak changes in the characteristic length scale of structural relaxation. Even attempts to study glass transition in confinement did not bring any conclusive results (see, e.g., review [215]). Overall, the role of cooperativity and dynamic heterogeneity in glass transition remains unclear and is the focus of many current studies.

Elastic models have been also successful in the description of *τ_α_*(*T*) in many materials. The increase in the activation energy of the structural relaxation upon approaching *T_g_* is indeed comparable to the temperature increase in the shear modulus. Elastic models also provide clear connections to the properties of fast dynamics, including MSD on the picosecond time scale. The latter can also be a measure of the free volume and, in this way, provides a connection to free volume ideas. In contrast to the entropic approach, elastic models do not predict a divergence of the time scale, which seems to be consistent with most of the experimental data.

Although none of those three major approaches (entropy, elasticity, and free volume) provides complete and consistent descriptions for all the systems, each of them gives a good description for most of the experimental data. This suggests that there is an underlying physics that unites those three approaches, and this question has been discussed in several papers [83,167]. Some hybrid models, such as the one proposed by Mirigian and Schweizer [82,83,84,85,86,87,88], are trying to combine various approaches in one consistent picture and provide a unified description of the liquid dynamics in the entire temperature range from very high *T* down to *T_g_*.

In addition to cooperativity and dynamics heterogeneity, an even more general and important question is whether there is a divergence of the structural relaxation time scale at a finite *T* below *T_g_*. This question is crucial, because the existence of this divergence would suggest true underlying phase transitions. Unfortunately, the current experimental data do not provide an unambiguous answer, and the theoretical models suggest both scenarios. Nevertheless, it seems that the current consensus is shifting more towards no divergence of *τ_α_*(*T*) below *T_g_*.
